# Discovering non-associated pressure-sensitive plasticity models with EUCLID

**DOI:** 10.1186/s40323-024-00281-3

**Published:** 2025-01-18

**Authors:** Haotian Xu, Moritz Flaschel, Laura De Lorenzis

**Affiliations:** 1 Empa, Swiss Federal Laboratories for Material Science and Technology, Überlandstrasse 129, Dübendorf, 8600 Switzerland; 2 Department of Mechanical and Process Engineering, Institute for Mechanical Systems, ETH Zürich, Zürich, 8092 Switzerland

**Keywords:** Full-field data, Model discovery, Sparse regression, Pressure-sensitive plasticity, Non-associated flow rule

## Abstract

We extend (EUCLID Efficient Unsupervised Constitutive Law Identification and Discovery)—a data-driven framework for automated material model discovery—to pressure-sensitive plasticity models, encompassing arbitrarily shaped yield surfaces with convexity constraints and non-associated flow rules. The method only requires full-field displacement and boundary force data from one single experiment and delivers constitutive laws as interpretable mathematical expressions. We construct a material model library for pressure-sensitive plasticity models with non-associated flow rules in four steps: (1) a Fourier series describes an arbitrary yield surface shape in the deviatoric stress plane; (2) a pressure-sensitive term in the yield function defines the shape of the shear failure surface and determines plastic deformation under tension; (3) a compression cap term determines plastic deformation under compression; (4) a non-associated flow rule may be adopted to avoid the excessive dilatancy induced by plastic deformations. In contrast to traditional parameter identification methods, EUCLID is equipped with a sparsity promoting regularization to restrain the number of model parameters (and thus modeling features) to the minimum needed to accurately interpret the data, thus achieving a compromise between model simplicity and accuracy. The convexity of the learned yield surface is guaranteed by a set of constraints in the inverse optimization problem. We demonstrate the proposed approach in multiple numerical experiments with noisy data, and show the ability of EUCLID to accurately select a suitable material model from the starting library.

## Introduction

Data-driven techniques for material modeling have rapidly gained prominence within the field of computational solid mechanics, finding extensive application across diverse material types such as metals, geomaterials, polymers, powders, and more. Unlike conventional material modeling practices, where experimental results are matched by identifying material parameters appearing in predefined constitutive laws, data-driven methods offer the capability to either *bypass* or *surrogate* the development of material models in the classical sense. This innovation, in turn, presents a compelling advantage in averting modeling errors stemming from the inherent discord between the a priori assumptions made by conventional models and the authentic material behaviors they seek to emulate.

Notwithstanding the progress made, the presently available methods still pose challenges due to their data-hungry and black-box nature. The state-of-the-art techniques [7, 24, 26, 32, 35, 43, 50, 52, 58] are rooted in a supervised learning or curve-fitting setting. Hence, they all need a large amount of data consisting of stress–strain pairs, which can only be collected in simple mechanical experiments like uniaxial tension and compression tests, torsion tests or bending tests. Furthermore, the experimental measurement of stress tensors presents a formidable challenge, as force measurements offer only partial data in the form of boundary-averaged stress tensor projections. Although artificial training datasets with stress–strain pairs can be obtained from finite element (FE) simulations at the microscale, these simulations are computationally expensive especially for 3D problems, and microscale simulations require detailed knowledge of the material properties and topology at the microscale, which is rarely available. To tackle this problem, the data-driven identification method developed by [9, 38] forms the inverse problem to the data-driven model-free approach proposed by [35]. Furthermore, attempts have been made to use only displacement and global force data to train artificial neural networks (ANNs) that surrogate constitutive models [27, 31, 53, 54]. However, both the data-driven identification method and black-box neural network methods have in common that the stress–strain relations remain uninterpretable, which can imply substantial challenges in enforcing or validating the adherence to physical constraints.

Considering both the demand for labeled data and the issue of interpretability, dealing with path-dependent material behaviors, like plasticity, becomes even more challenging. This complexity arises because the stress state at a material point is not solely determined by its strain state but is also influenced by the historical evolution of that material point, typically characterized by internal variables in traditional approaches. Path-dependent problems have been tackled with constitutive-model-free approaches that formulate forward problems directly guided by the available data [3–5, 13, 23, 32, 51], or by surrogating the path-dependent constitutive behavior using ANNs [30, 36, 37, 39, 43, 47, 56, 59], support vector machines [29], or symbolic regression [2]. Others leverage machine learning tools to utilize the insights obtained from the data to refine material models established in traditional theories [33, 42]. As they operate under supervised learning, all these methods demand an extensive volume of labeled data during the training process, specifically in the form of stress–strain paths, comprising stress–strain couples at each time step for every conceivable loading scenario. In essence, they necessitate the collection of stress–strain increments for numerous, potentially infinite loading histories. This requirement renders comprehensive coverage of the sampling space nearly unattainable, rendering the supervised learning task infeasible. This holds true regardless of whether the data originates from experiments (where, as previously noted, even path-independent sampling poses significant challenges) or from multiscale simulations (whose computational demands, already formidable in the path-independent context, now escalate to orders of magnitude higher levels).

Against this background, we recently proposed an unsupervised discovery framework called EUCLID (Efficient Unsupervised Constitutive Law Identification and Discovery), that combines the advantages of data-driven methods and traditional modeling approaches, see [14] for an overview. EUCLID can automatically discover the target model from a large library of interpretable candidate material models, with the input of full-field displacements and global reaction forces instead of labeled stress–strain data pairs. Until now, EUCLID has been successfully demonstrated for hyperelasticity [15–17, 22, 34], viscoelasticity [41], pressure-insensitive associated plasticity [18–20], and generalized standard material models [21].

**Fig. 1:**
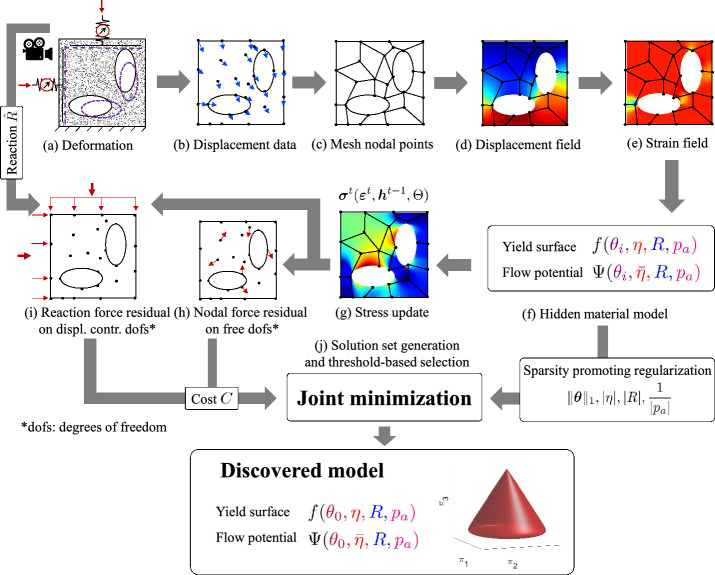
Step-by-step schematic of EUCLID. In a single experiment with complex geometry (**a**), point-wise displacements (**b**) and global reaction forces (i) are measured. A quadrilateral FE mesh is constructed (**c**) to interpolate the displacement data. The resulting displacement field (**d**) is differentiated to arrive at the strain field (**e**). The material model library (**f**) is constructed. Based on this library and for given material parameters $\varvec{\theta }$, $\eta$, $\bar{\eta }$, *R*, $p_a$, the stresses can be calculated by applying a classical elastic predictor - plastic corrector return mapping algorithm at each load step in the dataset, while the history variables are updated at each step (**g**). Based on the stresses, the internal and external virtual works and hence the internal (**h**) and external (**i**) force imbalances are calculated, contributing to the cost function *C*. Finally, the cost function is minimized jointly with a sparsity-promoting regularization term (**j**) to generate a set of solutions out of which a solution with low cost and high parsimony is automatically selected

Our objective with the present paper is to extend the flexibility of EUCLID in the context of plastic material model discovery. The first application of EUCLID presented in [18] is constrained to pressure-insensitive and associated plasticity models with isotropic and kinematic hardening, which provides a powerful framework for discovering constitutive models for metals. For other materials, like soils and concrete, the assumptions of pressure-insensitivity and associativity should be weakened, which is the main scope of this work. Additionally, the framework proposed in [18] allowed for the discovery of non-convex plastic yield surfaces. In this work, we restrain the yield surfaces to be convex, to ensure thermodynamic consistency. These goals are achieved by formulating an extensive material model library that comprises pressure-insensitive and associated plasticity, and by restraining the model parameters appropriately to ensure the yield surface convexity. From the material model library, and informed by full-field displacement and net reaction force data, EUCLID selects the most appropriate material model. The step-by-step schematic of EUCLID is illustrated in Fig. 1 and described in detail in “Unsupervised discovery of non-associated pressure-sensitive plasticity models” section. The framework is numerically verified for different test cases in “Numerical benchmarks” section and conclusions are drawn in “Conclusions” section.

Notation: Tensors and matrices may appear in compact or index notation, e.g., $\varvec{\sigma }$ or $\sigma _{ij}$, respectively. In compact notation, first-order tensors (vectors) and second-order tensors are expressed by bold letters, e.g., $\varvec{\sigma }$, and higher-order tensors by blackboard bold letters, e.g., $\mathbb {C}$. When appearing in index notation, the order of the tensor equals the number of the indices. If not stated otherwise, the Einstein convention for summation over repeated indices is used in equations appearing in index notation, e.g., $\sigma _{ij}n_j=\sum _j \sigma _{ij}n_j$. Indices separated by a comma denote partial derivatives, $u_{i,j}=\frac{\partial u_{i}}{\partial x_j}$. Inner products are denoted by $\cdot$, e.g., $\varvec{a}\cdot \varvec{b}= a_i b_i$, and outer products by $\otimes$, e.g., $(\varvec{a}\otimes \varvec{b})_{ij} = a_i b_j$. If no operation is indicated between two tensors, the juxtaposition implies tensor contraction, e.g., $(\varvec{\sigma }\varvec{n})_i=\sigma _{ij}n_j$. A colon denotes double contraction, e.g., $(\mathbb {C}:\varvec{\varepsilon })_{ij}=\mathbb {C}_{ijkl}\varepsilon _{kl}$. The trace of a tensor is denoted by $\text {tr}(\cdot )$, e.g., $\text {tr}(\varvec{\sigma }) = \sigma _{ii}$, the volumetric part by $\text {vol}(\cdot )$, the deviatoric part by $\text {dev}(\cdot )$, and the determinant by $\text {det}(\cdot )$.

## Unsupervised discovery of non-associated pressure-sensitive plasticity models

### Material model library

At the core of EUCLID stands a material model library, i.e., a set of potential candidate material models, in which EUCLID searches for a mathematically simple material model that is suited to describe the given data. In this section, we construct a model library that contains a variety of material models known from the classical theory of elastoplasticity (see, e.g., [10, 48]). We focus on homogeneous, isotropic materials with linear elastic behavior before plastic yielding. For the plastic behavior, we consider both pressure-sensitive and pressure-insensitive as well as associated and non-associated models in the library. We assume small-strain conditions. We ignore any hardening or softening behavior, which means that perfect plasticity is considered in this manuscript. For an existing demonstration of the discovery of hardening effects in the framework of EUCLID, the reader is referred to [18].

Following the classical theory of elastoplasticity, the total strain $\varvec{\varepsilon }$, as derived from the symmetric part of the spatial gradient of the displacement field $\varvec{u}$, i.e., $\varepsilon _{ij}=\frac{1}{2}(u_{i,j}+u_{j,i})$, is decomposed into the sum of an elastic component and a plastic component $\varvec{\varepsilon }=\varvec{\varepsilon }_e+\varvec{\varepsilon }_p$, where the plastic component $\varvec{\varepsilon }_p$ is a history variable. The constitutive law for the Cauchy stress is written as $\varvec{\sigma }=\mathbb {C}:\varvec{\varepsilon }_e$, where $\mathbb {C}$ is the fourth-order elastic stiffness tensor. The yield surface, which describes the frontier of the elastic domain in the stress space, is defined by the zero level set of the yield function $f(\varvec{\sigma })$. Further, the evolution of the plastic strain is determined through the plastic evolution law

1 $$\begin{aligned} \dot{\varvec{\varepsilon }}_p = \dot{\gamma }\frac{\partial \Psi (\varvec{\sigma })}{\partial \varvec{\sigma }}, \end{aligned}$$

where the superposed dot denotes the derivative with respect to time, and $\Psi (\varvec{\sigma })$ is the flow potential whose derivative determines the direction in which the plastic strain evolves. The model is called associated if $\Psi = f$, and non-associated otherwise. The plastic multiplier $\gamma$ and the yield function $f(\varvec{\sigma })$ need to fulfill the Karush-Kuhn-Tucker loading/unloading conditions, i.e., the constraints

2 $$\begin{aligned} f(\varvec{\sigma }) \leqslant 0, \quad \dot{\gamma }\geqslant 0, \quad \dot{\gamma }\ f(\varvec{\sigma }) = 0. \end{aligned}$$

**Fig. 2:**
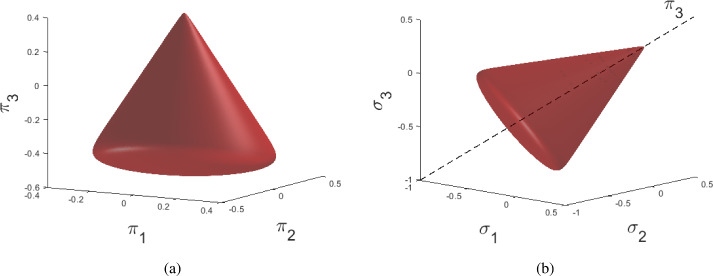
Exemplary yield surface plot **a** in the $\pi$-space and **b** in the principal stress space

As the material model library determines the space in which EUCLID searches for a material model in the inverse problem, we aim for our library to be as comprehensive as possible, covering many different potential candidate models. In this work, a versatile model library is constructed by suitable parameterizations of the material’s yield function *f* and flow potential $\Psi$. In the following, we first introduce the chosen model library and then discuss each individual component in detail. On the basis of the pressure-insensitive yield function parameterization proposed by [18], our pressure-sensitive yield function library reads

3 $$\begin{aligned} f(r,\alpha ,\pi _3)= &   \sqrt{\eta ^2 a^2 \left( \sum _{i=0}^{n_f}{\theta _i \cos (3i\alpha )}\right) ^2 + \frac{3}{2} r^2}- \left( 1 - \frac{1}{\sqrt{3}}\eta \pi _3 - e^{- R (\frac{1}{\sqrt{3}}\pi _3 - p_a)}\right) \nonumber \\  &   \sum _{i=0}^{n_f}{\theta _i \cos (3i\alpha )}\nonumber \\ \end{aligned}$$

where $\theta _i$, $\eta$, *R*, and $p_a$ are tunable material parameters, $n_f$ is an integer and *a* is a numerical parameter determining the smoothness of the yield surface. The Lode radius *r* and the Lode angle $\alpha$ are invariants of the stress tensor $\varvec{\sigma }$ which are here expressed in the so-called $\pi$-coordinate system (a rotated version of the coordinate system spanned by the principal stresses $\sigma _i$, as shown in Fig. 2)

4 $$\begin{aligned} r= &   \sqrt{\pi _1^2 + \pi _2^2}, \quad \alpha = \text {atan2}(\pi _1,\pi _2),\quad \text {with} \end{aligned}$$

5 $$\begin{aligned} \pi _1= &   \sqrt{\frac{2}{3}}\sigma _1 - \sqrt{\frac{1}{6}}\sigma _2 - \sqrt{\frac{1}{6}}\sigma _3, \quad \pi _2 = \sqrt{\frac{1}{2}}\sigma _2 - \sqrt{\frac{1}{2}}\sigma _3, \quad \pi _3 = \frac{1}{\sqrt{3}}(\sigma _1 + \sigma _2 + \sigma _3),\nonumber \\ \end{aligned}$$

where $\text {atan2}(\cdot ,\cdot )$ is the four-quadrant inverse tangent. Note that the third axis of the $\pi$-coordinate system is proportional to the hydrostatic pressure *p*, namely $p=\frac{1}{\sqrt{3}}\pi _3$.

As we do not want to limit ourselves to associated plasticity models in the library, we assume a different model ansatz for the flow potential than for the yield function, i.e., we parameterize the flow potential as

6 $$\begin{aligned} \Psi (r,\alpha ,\pi _3)= &   \sqrt{\bar{\eta }^2 a^2 \left( \sum _{i=0}^{n_f}{\theta _i \cos (3i\alpha )}\right) ^2 + \frac{3}{2} r^2}- \left( 1 - \frac{1}{\sqrt{3}}\bar{\eta } \pi _3 - e^{- R (\frac{1}{\sqrt{3}}\pi _3 - p_a)}\right) \nonumber \\  &   \sum _{i=0}^{n_f}{\theta _i \cos (3i\alpha )}, \end{aligned}$$

where we introduced an additional tunable material parameter $\bar{\eta }$. Given the yield function and the flow potential, the evolution of the variables at a material point [see Eq. (1)] can be computed through a return mapping algorithm, which is discussed in detail in Appendix A.

**Fig. 3:**
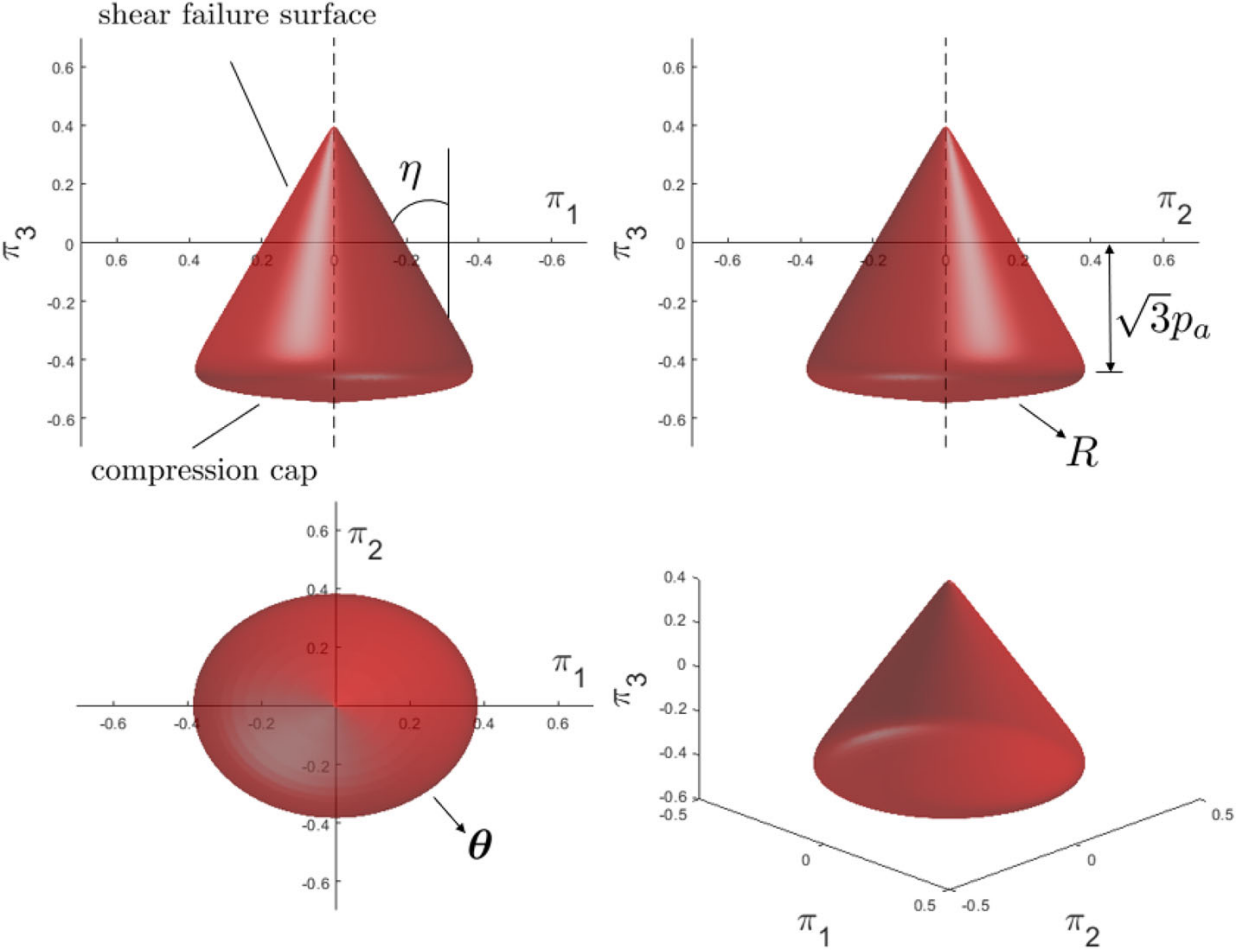
Effect of different material parameters on the shape of the yield surface in the $\pi$-space shown in different orthographic views

**Fig. 4:**
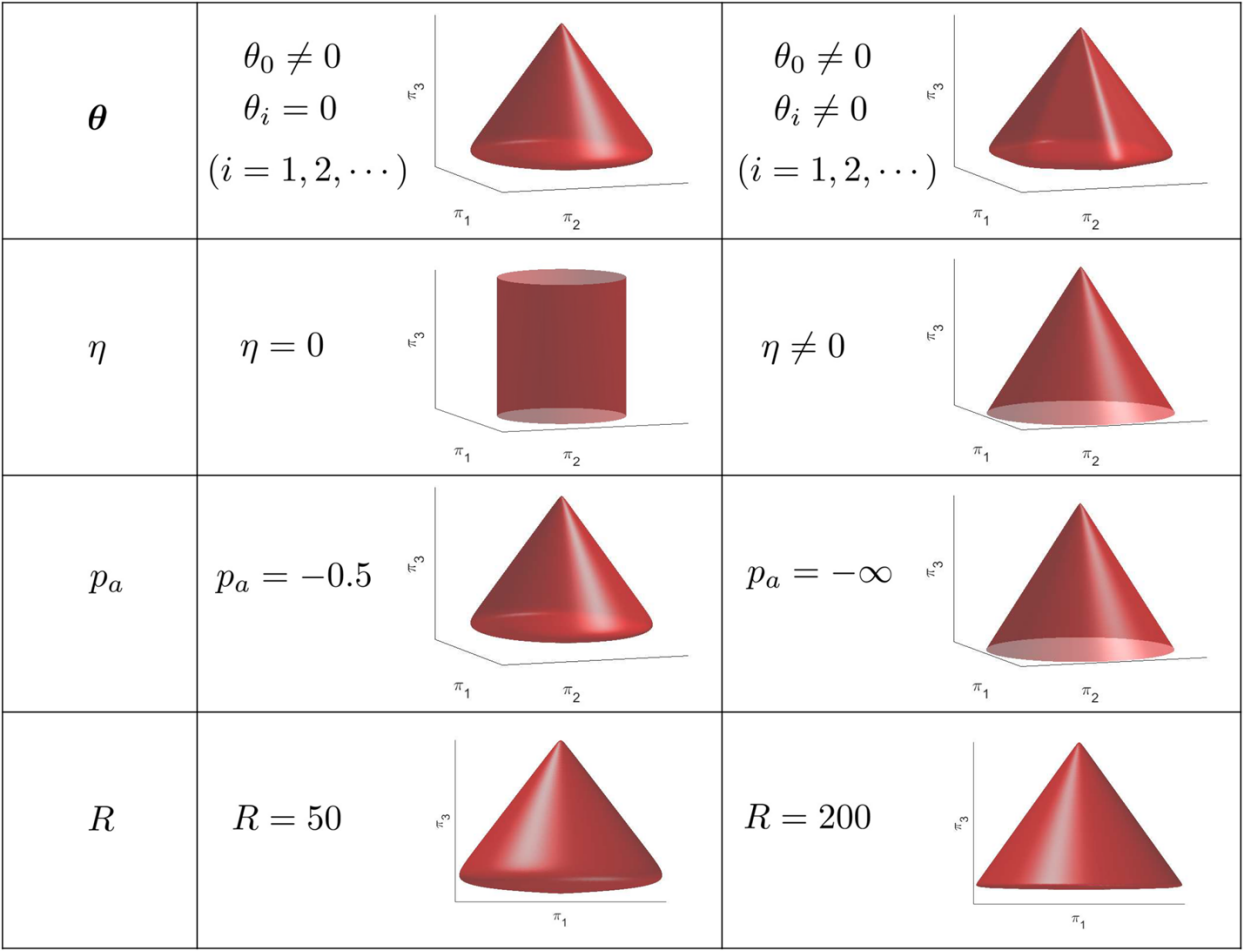
Effect of different material parameters on the shape of the yield surface in the $\pi$-space. Starting from an initial instantiation of parameters ($\theta _0\ne 0, \theta _i = 0, \eta \ne 0, p_a = -0.5, R = 50$), each parameter is varied individually to observe the effect on the yield surface

The material model library defined in Eqs. (3) and (6) includes a variety of elastoplastic material modeling features with different material model parameters. To ease a physical interpretation of the material model parameters, Fig. 3 shows in different orthographic views which parts of the yield surface are affected by which parameters, and Fig. 4 illustrates the effect on the yield surface upon changing these parameters. In the following, the different material modeling features and the meaning of the respective parameters are explained in detail:

- Arbitrary yield surface shape in the $\pi$-plane: Following [18], a Fourier series ansatz [see the summation terms in Eq. (3)] is used to describe the shape of the yield surface in the $\pi$-plane, i.e., the plane spanned by $\pi _1$ and $\pi _2$, as shown in the $\pi _1$-$\pi _2$ orthographic view of Fig. 3. The parameters $\theta _i$ are $(n_f+1)$ material parameters (with $n_f$ as a tailorable number) that govern the shape of the yield surface in the $\pi$-plane. By choosing a sufficiently large number of features in the Fourier series, any smooth shape of the yield surface can be described in the $\pi$-plane. In this work, however, we restrict the number of features in the Fourier series to a maximum of five (i.e., $n_f = 4$) leading to a sufficiently high expressiveness of the model library. Certain choices of material parameters in Eq. (3) lead to a non-convex yield function *f* and a non-convex shape of the elastic domain in the $\pi$-plane (see the model ”NC” in [18]). The non-convexity of the yield function can lead to numerical issues when solving the local problem, and to thermodynamic inconsistency [25, 49]. Therefore, we add a convexity constraint to our model library both in the forward (data generation) and inverse (EUCLID) problems. The constraint on the parameters $\theta _i$ is written as
  7 $$\begin{aligned} f \text { is convex} \Longleftrightarrow \theta _0 + \sum _{i=0}^{n_f}(1 + 9 i^2)\theta _i \cos (3 i \alpha ) \ge 0, \quad \forall \alpha \in [0,\frac{\pi }{3}]. \end{aligned}$$
  More details on its derivation and implementation are provided in Appendix B.
- Pressure sensitivity: For pressure-sensitive material models, the influence of the hydrostatic pressure on yielding may be significant. Following popular pressure-sensitive plastic models, like the Drucker–Prager model [11, 12] and the Mohr-Coulomb model [44, 57], which assume that the value of the yield function is linearly dependent on the hydrostatic pressure, we add a linear hydrostatic pressure term ($\eta \pi _3$ and $\bar{\eta }\pi _3$) to the yield function and flow potential [see Eqs. (3), (6)]. The material parameter $\eta \ge 0$, which is related to the friction angle of the material, defines the angle of the resulting conical part of the yield surface (usually called the shear failure surface), as shown in the $\pi _1$–$\pi _3$ orthographic view of Fig. 3. With $\eta > 0$, the term $\eta \pi _3$ delays yielding upon decreasing the value of $\pi _3$. The special case $\eta = 0$ recovers a pressure-insensitive material model (as in [18]), where the yield function is not dependent on $\pi _3$ and the yield surface takes the form of a cylinder or a prism, as shown in the second row of Fig. 4.
- Compression cap: Many materials show plastic deformation upon compression, e.g., for sufficiently small values of $\pi _3$, which is typically modeled by closing the yield surface in the compressive regime with a so-called compression cap. The compression cap is realized in our model by introducing an exponential function $e^{- R (\frac{1}{\sqrt{3}}\pi _3 - p_a)}$ in Eqs. (3), (6). This function changes the shape of the yield surface from an open-end surface in negative $\pi _3$-direction, i.e., a surface without a compression cap (as shown in the third row of Fig. 4 when $p_a=-\infty$), to a closed surface (as shown in Fig. 4 when $p_a=-0.5$). The reason for using an exponential term is that we desire an arc-shaped compression cap like in other yield surface models with compression caps (see, e.g., [6, 8, 28]) and do not want the new term to significantly affect the shape of the shear failure surface in the tensile regime. In contrast to other yield surface models with compression caps in the literature, which are typically multi-surface models, our model is a single-surface model without sharp corners which eases the application of the return mapping algorithm.
  $p_a$ (with $-\infty<p_a<0$) is a material parameter that defines the position of the compression cap, see the $\pi _2$-$\pi _3$ orthographic view of Fig. 3. In the limit $p_a\rightarrow -\infty$, the compression cap vanishes, as shown in the third row of Fig. 4. *R* (with $1 \leqslant R \leqslant 200$) is a material parameter that controls the shape of the compression cap. The larger *R*, the flatter the compression cap, as shown in the fourth row of Fig. 4. For a very small value of R, the shape of the compression cap becomes close to conical, whereas it is desirable to have an ellipsoidal surface such as in the multi-surface Drucker–Prager cap model [8, 28], and for this reason we enforce for *R* a lower bound of 1. When *R* is larger than 200, the transition between the shear failure surface and the compression cap becomes so sharp that it may negatively affect the solution process of the local problem.
- Smooth yield surface: The yield function library proposed in Eq. (3) is a smooth approximation of
  8 $$\begin{aligned} f(r,\alpha ,\pi _3)=\sqrt{\frac{3}{2}} r-\left( 1 - \frac{1}{\sqrt{3}}\eta \pi _3 - e^{- R (\frac{1}{\sqrt{3}}\pi _3 - p_a)}\right) \sum _{i=0}^{n_f}{\theta _i \cos (3i\alpha )}. \end{aligned}$$
  While the yield function library above would include all the desired modeling features (an arbitrarily shaped yield surface in the $\pi$-plane, pressure sensitivity, and compression cap), its corresponding yield surface is non-smooth at two points on the $\pi _3$-axis. To avoid working with sub-differentials in the return mapping method as needed for models like Drucker–Prager and Mohr–Coulomb [10], we follow [1] and use a differentiable hyperbolic surface to approximate $\sqrt{\frac{3}{2}} r$ in Eq. (8) with the hyperbolic term $\sqrt{\eta ^2 a^2 \left( \sum _{i=0}^{n_f}{\theta _i \cos (3i\alpha )}\right) ^2 + \frac{3}{2}r^2}$ [see Eq. (3)]. The user-defined parameter *a* controls the discrepancy between the non-smooth surface and its smooth approximation. The non-smooth surface is recovered for $a\rightarrow 0$. In contrast to the other parameters (i.e., $\varvec{\theta }$, $\eta$, *R*, and $p_a$), *a* is a purely numerical parameter and it is chosen as $a=0.01$ throughout this paper.
- Non-associativity: Normally, a non-associated flow rule is used for pressure-sensitive material models to avoid excessive plastic volume change, i.e., overestimated dilatancy. To introduce non-associativity into our model, we define a new parameter $\bar{\eta }$ as a substitute of $\eta$ in the flow potential. In general, $\bar{\eta }$ is used to decrease the dilatancy prediction, therefore, usually it is assumed that $0\le \bar{\eta }\le \eta$. However, here we loosen this constraint during the inverse analysis.

As the elastic material behavior is assumed to be linear and the elastic material properties can be determined during the linear loading part of a mechanical experiment, we assume that the elastic stiffness tensor $\mathbb {C}$ is already known, and we only need to discover the plastic components of the material model. We denote as $\Theta =\{\varvec{\theta },\eta ,\bar{\eta }, R, p_a\}$ all trainable material parameters in the inverse problem. These parameters govern the stress update procedure needed both for the forward FE simulations (used to generate the artificial data) and for the inverse discovery algorithm (EUCLID). That is, given the strain $\varvec{\varepsilon }^t$ at the current time step *t*, the history variables $\varvec{h}^{t-1} = \{\varvec{\varepsilon }^{t-1}_{p}, \gamma ^{t-1}\}$ of the previous time step and the material parameters $\Theta$, the current stress $\varvec{\sigma }^t(\varvec{\varepsilon }^{t}, \varvec{h}^{t-1}, \Theta )$ is calculated via a classical elastic predictor-plastic corrector return mapping algorithm (see Appendix A).

### Available data

The objective of EUCLID is to select a suitable material model from the previously introduced model library, relying on full-field displacement data, e.g. provided by digital image correlation (DIC) measurements, and net reaction force data. We assume a 2D specimen with measured displacements $\left\{ \varvec{u}^{a,t}:a=1,...,n_n;t=1,...,n_t\right\}$ at $n_n$ points on the specimen surface over $n_t$ time steps, as well as a number of $n_{\beta }$ measured reaction force components $\left\{ \hat{R}^{\beta ,t}:\beta =1,...,n_{\beta };t=1,...,n_t\right\}$. To obtain continuous displacement fields, the nodal displacement data are interpolated using a FE mesh with shape functions $\{N^a(\varvec{X}):a=1,\dots ,n_n\}$, i.e., $\varvec{u}^{t}(\varvec{X}) = \sum _a N^a(\varvec{X}) \varvec{u}^{a,t}$. The strain fields at each load step can then be obtained by differentiating the displacement fields, i.e., $\varvec{\varepsilon }^{t}(\varvec{X}) = \sum _a \text {sym}(\nabla N^a (\varvec{X}) \otimes \varvec{u}^{a,t})$.

### Optimization problem

In contrast to supervised learning strategies, EUCLID does not rely on labeled stress–strain data pairs. Instead, EUCLID is based on a physics-driven and thus unsupervised optimization problem. The objective is to identify a material model in the library such that the governing physical laws (in this case the balance of linear momentum) are satisfied. Neglecting body forces, the quasi-static balance of linear momentum over a body $\Omega$ with boundary $\partial \Omega$ in its weak form states that

9 $$\begin{aligned} \int _\Omega \varvec{\sigma }^{t}(\varvec{\varepsilon }^t,\varvec{h}^{t-1},\Theta ):\nabla \varvec{v}\;\!\textrm{d}A = \int _{\partial \Omega } \hat{\varvec{t}}^t\cdot \varvec{v}\;\!\textrm{d}s, \end{aligned}$$

must hold true for all admissible test functions $\varvec{v}$, where $\hat{\varvec{t}}^t$ denotes the traction at the boundary. Choosing the shape functions $N^a$ for discretizing the test functions, we obtain the following nodal internal forces in the FE sense

10 $$\begin{aligned} \varvec{F}^{a,t}(\Theta ) = \int _{\Omega } \varvec{\sigma }^{t}(\varvec{X},\varvec{\varepsilon }^t,\varvec{h}^{t-1},\Theta ) \nabla N^a(\varvec{X}) dA. \end{aligned}$$

Equilibrium dictates that the nodal internal forces should vanish at all free degrees of freedom, and the nodal internal forces at the boundary should be equal to the measured reaction force. Denoting the set of free degrees of freedom as $\mathcal {D}^{\text {free}}$, we define the cost function

11 $$\begin{aligned} C^\text {free}(\Theta ) = \sum _{t=1}^{n_t}\sum _{(a,i) \in \mathcal {D}^\text {free}} \left| F^{a,t}_i(\Theta )\right| ^2. \end{aligned}$$

Further, we denote as $\mathcal {D}^{\text {disp},\beta }$ the set of the degrees of freedom that correspond to the boundary at which a measured reaction force $\hat{R}^{\beta ,t}$ is given, and we define another cost function

12 $$\begin{aligned} C^\text {disp}(\Theta ) = \sum _{t=1}^{n_t} \sum _{\beta =1}^{n_\beta } \left| \hat{R}^{\beta ,t} - \sum _{(a,i)\in \mathcal {D}^{\text {disp},\beta } } F^{a,t}_i(\Theta ) \right| ^2. \end{aligned}$$

Finally, we combine the two costs in a single cost function

13 $$\begin{aligned} \begin{gathered} C(\Theta ) = C^{\text {free}}(\Theta ) + \lambda _r C^{\text {disp}}(\Theta ), \end{gathered} \end{aligned}$$

where $\lambda _r>0$ is a balancing hyperparameter used to control the contribution of $C^{\text {disp}}$ to the total cost function. We choose $\lambda _r = 100$ and keep it constant throughout the numerical experiments, as in the previous study on pressure-insensitive plasticity [18].

Because of the expressive material model library with a large number of possible combinations of active modeling features, minimizing the cost function in Eq. (13) is highly ill-posed, and it would in general result in a dense solution vector $\Theta$. To obtain a sparse and thus interpretable solution, we add an $\ell _1$-norm-inspired regularization term to the cost function to arrive at the regularized and constrained minimization problem

14 $$\begin{aligned} \begin{gathered} \Theta ^{\text {opt}} = {{\,\mathrm{{arg\,min}}\,}}_{\Theta } \left\{ C(\Theta ) + \lambda _p\left( \sum _{i=1}^{n_f}|\theta _i| + \lambda _{\eta } |\eta | + \lambda _R |R| + \lambda _{p_a}\frac{1}{|p_a|}\right) \right\} , \\ \text {s.t.} \quad \text {{ f} is convex (Eq.~7)},~ \eta \ge 0,~ \bar{\eta } \ge 0,~ 1 \le R \le 200,~ p_a \le 0. \end{gathered} \end{aligned}$$

The regularization term—which is inspired by the LASSO (least absolute shrinkage and selection operator) [55]—penalizes the absolute values of the parameters $\theta _i$, $\eta$ and *R*, and the reciprocal of the absolute value of the parameter $p_a$. In this way, the regularized optimization problem favours solutions with vanishing parameters, and thus simpler models over complex models. If many of the parameters $\theta _i$ are zero, the material model simplifies because the shape of the yield surface in the $\pi$-plane simplifies. If $\eta$ is zero, the material model simplifies because the linear pressure-sensitive term vanishes. Finally, if the reciprocal of $p_a$ tends to zero, the material model simplifies because the compression cap vanishes. Note further that the choice of the parameter *R* becomes indifferent if the compression cap vanishes. Thus, a penalty on the parameter *R* is added to the minimization problem to increase the numerical efficiency and counteract solution non-uniqueness. The hyperparameter $\lambda _p>0$ balances the contribution of the regularization term to the total cost function, and $\lambda _{\eta }>0$, $\lambda _{R}>0$ and $\lambda _{p_a}>0$ are three additional hyperparameters used to scale the influence of the regularization on the respective material parameters. An optimal strategy for choosing these hyperparameters is discussed in the next section.

### Solution strategy

The problem in Eq. (14) describes a non-convex constrained optimization problem. The convexity constraint [see Eq. (7)] should be fulfilled for all possible choices of the Lode angle $\alpha$. Here, we slightly relax this constraint and assume that it is sufficient if Eq. (7) is fulfilled for a large but finite number of Lode angles (see Appendix B for details). In this way, the problem simplifies to a non-convex optimization problem with linear constraints. Such problems can be efficiently tackled with the solver *fmincon* provided in Matlab^®^.

Due to its non-convexity, the objective function in Eq. (14) may exhibit multiple local minima. To increase the chance of finding the global minimum (or a sufficiently low local minimum) of the objective function, we first solve the unregularized version of Eq. (14) (i.e., $\lambda _p=0$) for multiple randomly chosen initial guesses. In particular, we choose $n_g = 100$ initial random guesses. From the resulting $n_g = 100$ solutions, we choose the one with the lowest objective function value and discard the others. We then assume that the chosen solution is sufficiently close to the global minimum of the regularized objective function (i.e., $\lambda _p \ne 0$), and thus choose it as the initial guess for solving the regularized problem.

Before solving the regularized problem, an assumption on the values of $\lambda _{\eta }$, $\lambda _{R}$, and $\lambda _{p_a}$ has to be made. To properly scale the individual contributions to the regularization term, we set $\lambda _{\eta }$, $\lambda _{R}$ and $\lambda _{p_a}$ such that the initial guess for the parameters (which we obtained from the unregularized optimization problem) satisfies $\Vert \varvec{\theta }\Vert _{1} = \lambda _{\eta } \eta = \lambda _R R = \lambda _{p_a}\frac{1}{|p_a|}$. In this way, the influence of the different regularization terms on the optimization problem is expected to be similar.

Finally, it is necessary to find an optimal choice for $\lambda _p$. The overarching goal is to achieve a compromise between model accuracy and simplicity. To this end, we perform a Pareto analysis, as explained in [18]. In particular, we solve Eq. (14) for a set of different values of $\lambda _p$. Specifically, we set $\lambda _p\in \{2^i: i = -9,...,20 \}$. From the resulting solutions, we discard those that exhibit a poor fitting accuracy, i.e., the solutions for which the unregularized cost *C* is larger than the threshold $C^{th}$. The threshold is chosen such that $C^{th}=1.0001 \cdot C^{min}$ where $C^{min}$ is the lowest unregularized cost observed for all solutions obtained from the regularized optimization problem. From the remaining low-cost solutions, we select the sparsest solution, i.e., the solution with the smallest regularization term $\left( \sum _{i=1}^{n_f}|\theta _i| + \lambda _{\eta } |\eta | + \lambda _R |R| + \lambda _{p_a}\frac{1}{|p_a|}\right)$. Finally, to further enhance the model sparsity, any material parameter $\theta _i$ that is less than a threshold $\theta ^{th}$ is set to zero, as well as $\eta$ if it falls below a threshold $\eta ^{th}$ for pressure-insensitive models (same for $\bar{\eta }$ with threshold $\bar{\eta }^{th}$). Besides, $p_a$ is set to $-\infty$ if it is below the threshold $p_a^{th}$ for models without compression cap, and at the same time, *R* is set to ”Not a Number” (NaN) since it is meaningless when there is no compression cap. This strategy ensures that the final model is both accurate and characterized by a minimal number of parameters. We refer to Table 4 in Appendix D for a list of hyperparameters used during the inverse problem.

## Numerical benchmarks

### Data generation

Twelve different elasto-plastic material models are chosen to test EUCLID. The material parameters of the models are displayed in Tables 1 and 2 and the following list provides the model names, their abbreviations, and the references to the figures where the corresponding yield surfaces are illustrated:

- DP*: Hyperbolic approximation of the Drucker–Prager yield function (see Figs. in Appendix F).
- TR*P: Convex and smooth approximation of the pressure-dependent Tresca yield function (see Fig. 7a).
- IV*P: Convex and smooth approximation of the pressure-dependent Ivlev yield function (see Figs. in Appendix F).
- MA*P: Convex and smooth approximation of the pressure-dependent Mariotte yield function (see Fig. 7b).
- DP*C: Hyperbolic approximation of the Drucker–Prager model with compression cap with single-surface yield function (see Fig. 7c).
- TR*PC: Convex and smooth approximation of the pressure-dependent Tresca model with compression cap with single-surface yield function (see Fig. 8a).
- IV*PC: Convex and smooth approximation of the pressure-dependent Ivlev model with compression cap with single-surface yield function (see Fig. 8b).
- MA*PC: Convex and smooth approximation of the pressure-dependent Mariotte model with compression cap with single-surface yield function (see Fig. 8c).
- VM: Von-Mises yield function (see Fig. 9a).
- TR*: Convex and smooth approximation of the Tresca yield function (see Figs. in Appendix F).
- IV*: Convex and smooth approximation of the Ivlev yield function (see Fig. 9b).
- MA*: Convex and smooth approximation of the Mariotte yield function (see Fig. 9c).

Some of the models that are considered here, i.e., those attributed to Tresca, Ivlev and Mariotte, are originally described by non-smooth yield surfaces. As mentioned earlier, in this work we avoid non-smooth yield surfaces to avoid working with sub-differentials in the return mapping method. Instead, we consider convex and smooth approximations of such models. To this end, we use the Fourier series, i.e., we adjust the values of the $\theta _i$ in Eq. (3) to approximate the Tresca, Ivlev and Mariotte models (see Appendix C for details). Due to the constraint that the yield surface should be convex, higher-order terms (i.e., highly oscillating terms) in the Fourier series vanish, which is the reason why the number of active features in the Fourier series does not exceed five for the benchmark material models. We denote the approximate models with a star to distinguish them from the original non-smooth models. Likewise, the Drucker–Prager models considered here are smooth approximations of the original models due to the hyperbolic approximation.

The 12 benchmark material models are chosen such that multiple different modeling aspects are represented. Four different shapes in the $\pi _1$–$\pi _2$-plane can be observed in the set of models. For each of these shapes, a pressure-sensitive model without compression cap, a pressure-sensitive model with compression cap, and a pressure-insensitive model are constructed. Thus the first four models (DP*, TR*P, IV*P, and MA*P) in the aforementioned list are pressure-sensitive models without compression cap, the next four models (DP*C, TR*PC, IV*PC, and MA*PC) are pressure-sensitive models with compression cap, and the last four models (VM, TR*, IV*, and MA*) are pressure-insensitive. Although all these models exhibit different modeling features, they can all be considered as specific instances of the material model library [see Eq. (3)]. For the pressure-sensitive models without compression cap, the parameter $p_a$ is chosen to take an extremely small value, such that the compression cap is so far away from the origin of the $\pi$-space that the models can be considered as models without compression cap, i.e., the yield surfaces are open in the negative $\pi _3$-direction. For the pressure-insensitive models, the parameter $p_a$ is extremely small and the parameters $\eta$ and $\bar{\eta }$ vanish, such that the compression cap vanishes and the shear failure surface becomes parallel to the $\pi _3$-coordinate, i.e., the yield surfaces are open in the positive and negative $\pi _3$-direction.

**Fig. 5:**
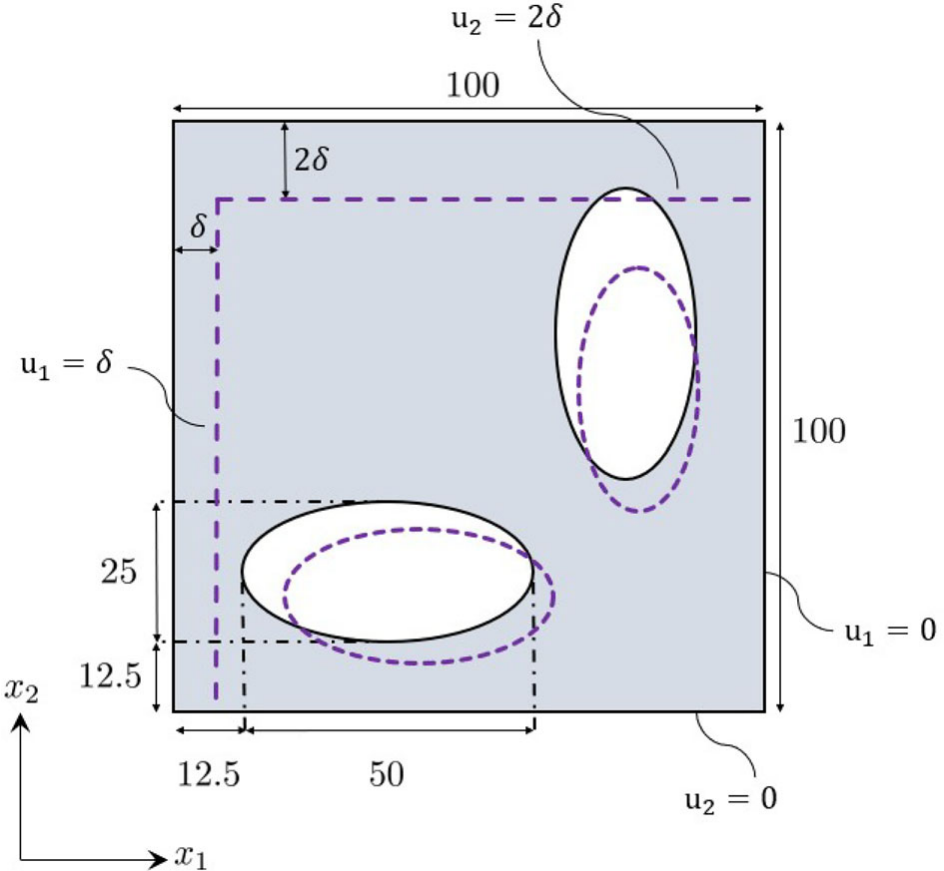
Geometry and boundary conditions of the chosen domain. All dimensions are in mm

**Fig. 6:**
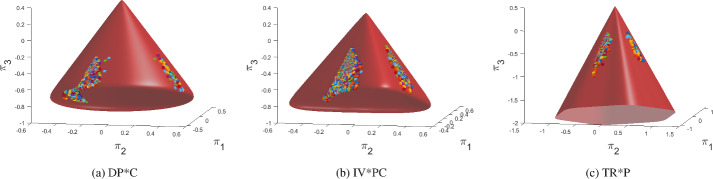
Examples of visualization on the yield surface of the data from the biaxial compression test in Fig. 5

EUCLID takes full-field displacements and global reaction forces as input, which can be obtained, e.g., from DIC and load cells, respectively. In this work, both the full-field displacement and the reaction force data are artificially generated with FE simulations based on the previously listed benchmark material models. The default parameters and hyperparameters used for FE data generation are summarized in Table 3 in Appendix D. In contrast to traditional methods for parameter calibration, which rely on simple testing setups, like uniaxial tension and compression tests, torsion tests or bending tests, for our application we desire a testing scenario in which the material experiences a diverse variety of deformation states. To this end, we adopt a biaxial compression test as shown in Fig. 5, for which we assume plane-strain conditions. To promote a complex deformation field over the specimen, two elliptical holes are introduced in the specimen geometry. In addition, we choose non-symmetric boundary conditions to further enhance the complexity of the dataset. We fix the displacement in $x_2$-direction of the bottom edge and the displacement in $x_1$-direction of the right edge, and then impose a displacement of $2\delta$ in $x_2$-direction to the top edge and a displacement of $\delta$ in $x_1$-direction value to the left edge. Specifically, we choose $\delta =0.1\,\text {mm}$ and apply the deformation over $n_t = 400$ load steps. For each load step, the displacements at all nodes in the FE mesh are recorded. In addition to the displacements, we record the total horizontal reaction forces on the left edge and vertical reaction forces on the top edge. We believe that the general quality of the dataset is highly affected by the coverage of the yield surface by the data points. Therefore, when we generate the numerical dataset, we also record the stress state of each quadrature point in the mesh and plot it on each yield surface. Examples of such visualization of the dataset are shown in Fig. 6, and the remaining plots are provided in Appendix E.

Independent Gaussian noise with zero mean and standard deviation $\sigma > 0$ is added to the FE displacement data to imitate the DIC data from real experiments. Three different levels of noise, including $\sigma = 0$ for the noiseless data, are applied to the displacement results, i.e., $\sigma \in \{0 \,\text {mm}, 1 \cdot 10^{-4}\, \text {mm}, 3 \cdot 10^{-4} \,\text {mm}\}$. A reasonable estimate for the measurement noise of modern DIC setups is usually considered as $\sigma = 0.1\,\upmu \text {m} = 1 \cdot 10^{-4}\, \text {mm}$ [40, 45]. A smoothing procedure is implemented afterwards, as it would be done in practice with experimental data, to decrease the influence of noise on the optimization results. We use the Savitzky–Golay filter [46] based on quadratic polynomial fitting, which is implemented in the *smoothdata* function in Matlab^®^, with a moving-window length of 20 time steps. Note that only temporal smoothing is applied to the data. To further increase the resistance to noise, spatial smoothing could be considered additionally.

### Results

EUCLID is applied to the datasets corresponding to the twelve benchmark material models and the three noise levels to discover the material response. The true and identified material parameters appearing in the discovered yield function and flow potential are summarized in Tables 1 and 2. To further assess the accuracy of the discovered models, 3D plots of true and discovered yield surfaces are provided. For the sake of brevity, only the yield surface plots for selected models corresponding to the intermediate noise level are shown in this section, whereas the remaining plots are provided in Appendix F.

**Table 1:** Parameters of true and discovered *pressure-sensitive* material models for different noise levels $\sigma$ (in mm)

Benchmarks	$\theta _0$	$\theta _1$	$\theta _2$	$\theta _3$	$\theta _4$	$\eta$	$\bar{\eta }$	*R*	$p_a$
DP*									
Truth	0.2400	0	0	0	0	4.1667	0.8333	NaN	$-\infty$
$\sigma = 0$	0.2400	0	0	0	0	4.1667	0.8333	NaN	$-\infty$
$\sigma = 10^{-4}$	0.2405	0	0	0	0	4.1486	0.8394	NaN	$-\infty$
$\sigma = 3 \cdot 10^{-4}$	0.2589	0	0	0	0	3.7084	0.8641	NaN	$-\infty$
TR*P									
Truth	0.2181	0	0.0095	0	0.0013	4.5861	0.9172	NaN	$-\infty$
$\sigma = 0$	0.2178	0	0.0084	0	0	4.5635	0.9137	NaN	$-\infty$
$\sigma = 10^{-4}$	0.2181	0	0.0067	0	0	4.5143	0.9389	NaN	$-\infty$
$\sigma = 3 \cdot 10^{-4}$	0.2438	0	0.0026	0	0	3.6300	0.9111	NaN	$-\infty$
IV*P									
Truth	0.1618	0.0300	0.0066	0.0021	0	6.1795	1.2359	NaN	$-\infty$
$\sigma = 0$	0.1588	0.0272	0.0052	0.012	0	6.2845	1.1952	NaN	$-\infty$
$\sigma = 10^{-4}$	0.1656	0.0261	0.0054	0.0010	0	5.8979	1.1249	NaN	$-\infty$
$\sigma = 3 \cdot 10^{-4}$	0.1711	0.0255	0.0054	0.009	0	5.7849	1.1416	NaN	$-\infty$
MA*P									
Truth	0.1954	−0.0332	0.0073	−0.0028	0.0012	5.1174	1.0235	NaN	$-\infty$
$\sigma = 0$	0.1932	−0.0326	0.0064	−0.0018	0	5.1580	1.0542	NaN	$-\infty$
$\sigma = 10^{-4}$	0.1956	−0.0328	0.0065	−0.0018	0	5.0532	1.0478	NaN	$-\infty$
$\sigma = 3 \cdot 10^{-4}$	0.2891	−0.0251	0.0108	0	0	3.4174	0.8713	NaN	$-\infty$
DP*C									
Truth	0.2400	0	0	0	0	4.1667	0.8333	50.0000	−0.5000
$\sigma = 0$	0.2400	0	0	0	0	4.1667	0.8333	50.0000	−0.5000
$\sigma = 10^{-4}$	0.2450	0	0	0	0	4.1667	0.8333	52.5300	−0.4999
$\sigma = 3 \cdot 10^{-4}$	0.1570	0	−0.0047	0.0010	0	10.4666	10.4653	8.5218	−0.3515
TR*PC									
Truth	0.2181	0	0.0095	0	0.0013	4.5861	0.9172	50.0000	−0.5000
$\sigma = 0$	0.2173	0	0.0084	0	0	4.5802	0.9150	51.0666	−0.5004
$\sigma = 10^{-4}$	0.2161	0	0.0083	0	0	4.6251	0.9193	57.3043	−0.4975
$\sigma = 3 \cdot 10^{-4}$	0.2474	0	0.0057	0	0	3.6356	0.9420	36.0818	−0.5233
IV*PC									
Truth	0.1618	0.0300	0.0066	0.0021	0	6.1795	1.2359	50.0000	−0.5000
$\sigma = 0$	0.1748	0.0245	0.0047	0.0013	0	5.2749	0.4143	NaN	$-\infty$
$\sigma = 10^{-4}$	0.1607	0.0215	0.0056	0.0009	0	6.1255	0.4517	NaN	$-\infty$
$\sigma = 3 \cdot 10^{-4}$	0.1962	0.0230	0.0067	0	0	4.3091	0.4013	NaN	$-\infty$
MA*PC									
Truth	0.1954	−0.0332	0.0073	−0.0028	0.0012	5.1174	1.0235	50.0000	−0.5000
$\sigma = 0$	0.1918	−0.0323	0.0064	−0.0018	0	5.2205	1.0587	53.1312	−0.5007
$\sigma = 10^{-4}$	0.1915	−0.0316	0.0064	−0.0018	0	5.2560	1.0590	55.6741	−0.4979
$\sigma = 3 \cdot 10^{-4}$	0.2229	−0.0334	0.0047	0	0	4.1400	0.9618	157.5327	−0.4931

$\varvec{\theta }$ are in $\text {kN/}\text {mm}^2$. $p_a$ is in Pa

**Fig. 7:**
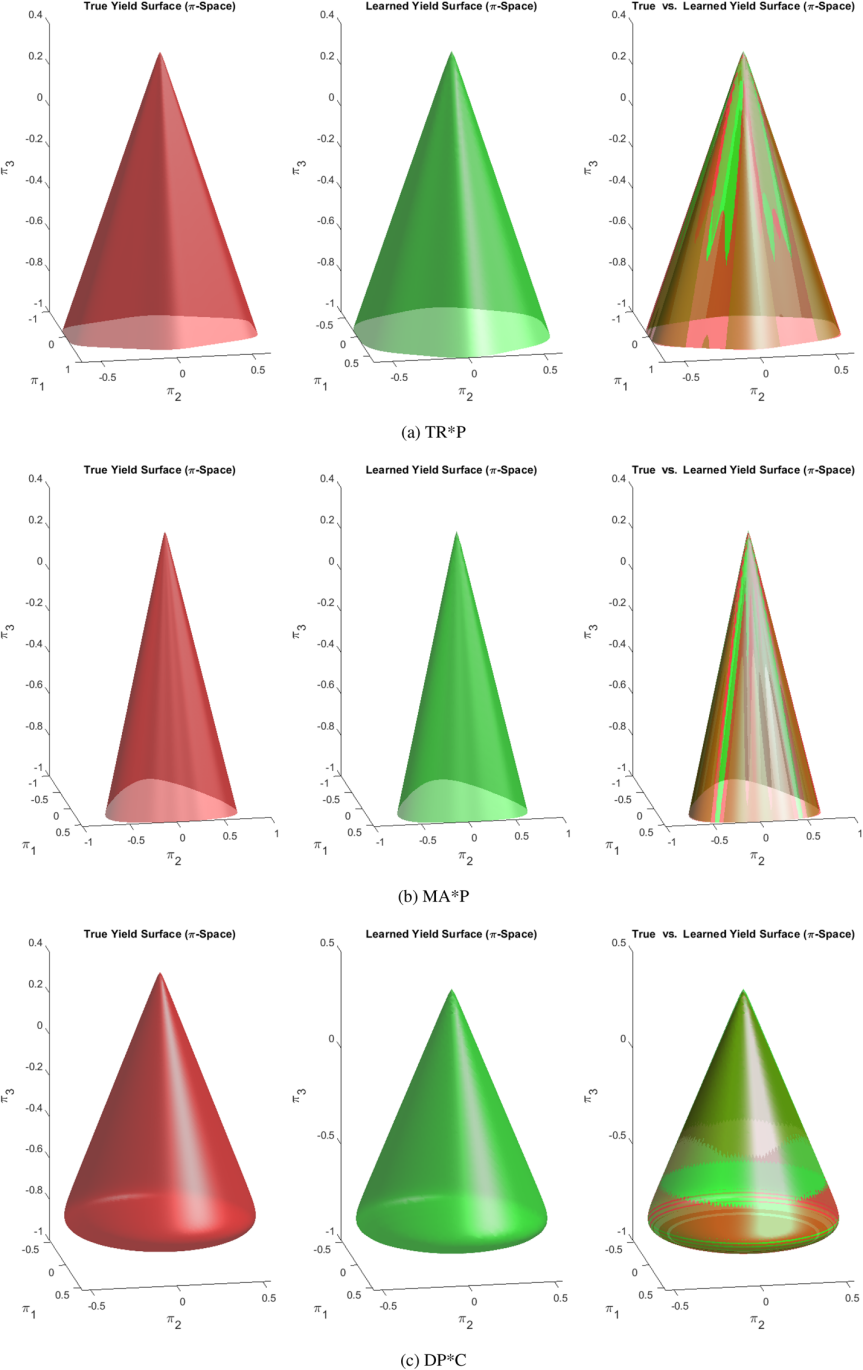
3D yield surface plots of the true and discovered *pressure-sensitive* plasticity models for the intermediate noise level ($\sigma = 10^{-4}$)

**Fig. 8:**
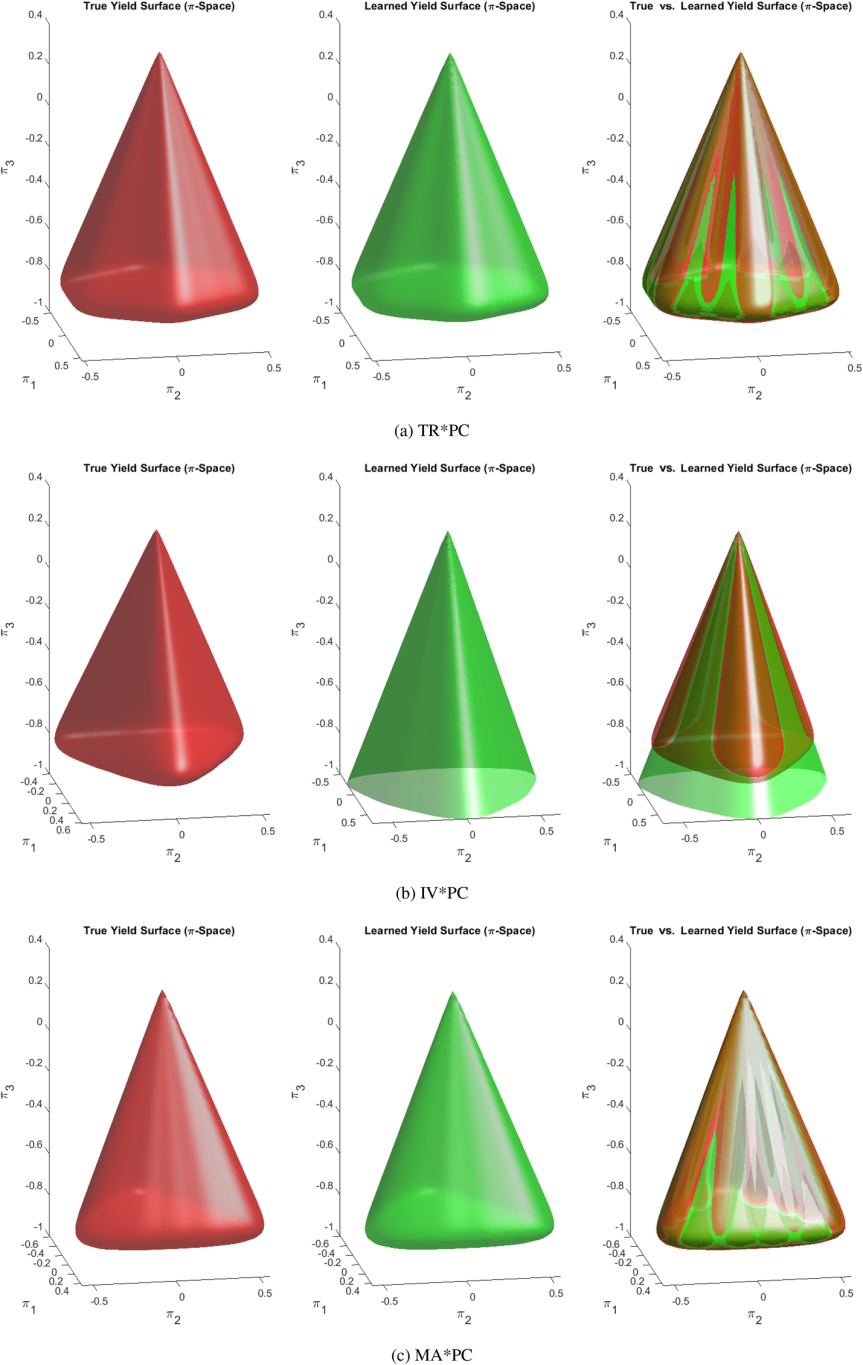
3D yield surface plots of the true and discovered *pressure-sensitive* plasticity models for the intermediate noise level ($\sigma = 10^{-4}$)

The material parameters for the true and discovered pressure-sensitive material models are shown in Table 1. In the noiseless case ($\sigma = 0$), the models DP* and DP*C are discovered exactly, while the other models are discovered with a very high accuracy. For the intermediate noise level ($\sigma = 10^{-4}$), the discrepancy between the true and discovered parameters slightly increases compared to the noiseless case. However, the discovered solutions for all eight models in Table 1 are still in good agreement with the ground truth. This is also reflected in the 3D plots of the true and discovered yield surfaces in Figs. 7 and 8. As expected, for the largest noise level ($\sigma = 3 \cdot 10^{-4}$), which is beyond the realistically expected measurement noise in DIC experiments, the discrepancy between the true and discovered parameters further increases. For most of the material models, the discovered models exhibit a larger value of $\theta _0$ and smaller values of $\theta _i$ $(i=1,2,3,4)$ compared to the ground truth. For the majority of the models shown in Table 1, EUCLID not only identifies the unknown parameters, but it is further able to learn which modeling features should be present in the discovered model, and which modeling features can be neglected. Only for some models, false-negative feature predictions (i.e., features that are not discovered although active in the true model) and false-positive feature predictions (i.e., features that appear in the discovered formula but are not present in the true model) are observed. Both false-negative and false-positive feature predictions increase upon increasing the noise, as expected. However, as can be observed in Figs. 7 and 8, most of the false feature predictions have a marginal influence on the fitting accuracy. An explanation for this could be that, due to the high expressiveness of the model library, there are different models in the library that exhibit similar material behavior. There is one exception for the model IV*PC. For this model, EUCLID makes a false-negative feature prediction for the compression cap. As shown in Fig. 8b, the true and discovered yield surfaces are very similar in the tensile region, but the discovered model diverges from the true model in the compressive region. A possible explanation for this could be that the dataset provided to EUCLID does not entail enough information in the compressive region, as shown in Fig. 6b.

**Fig. 9:**
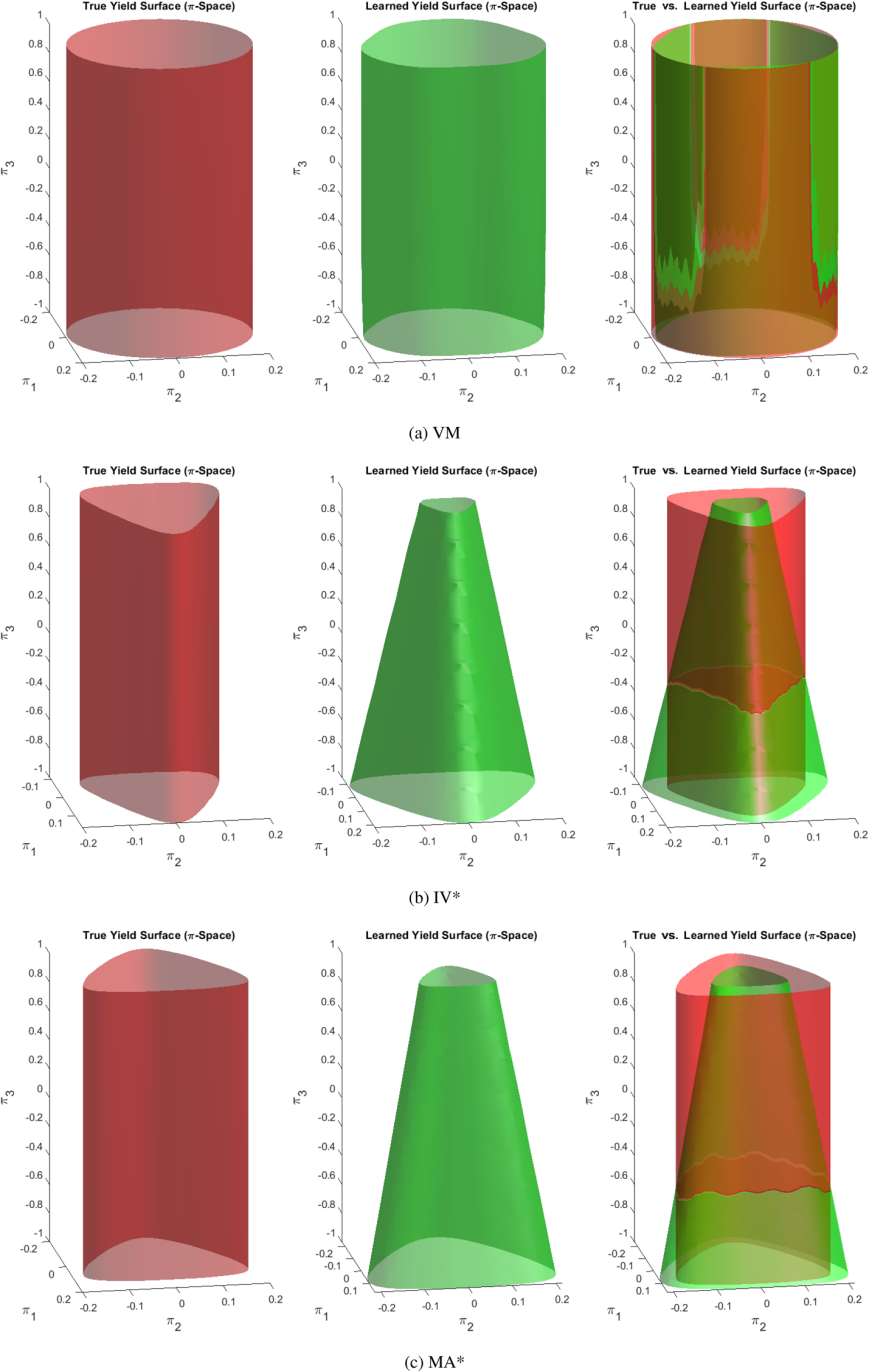
3D yield surface plots of the true and discovered *pressure-insensitive* plasticity models for the intermediate noise level ($\sigma = 10^{-4}$)

Table 2 shows the material parameters for the true and discovered pressure-insensitive material models. As for the pressure-sensitive models, upon increasing the noise level the accuracy of the identified parameters decreases and the number of false feature predictions increases. 3D plots of the true and discovered yield surfaces are shown in Fig. 9. EUCLID correctly identifies the shape of the yield surfaces in the $\pi _1$–$\pi _2$-plane. However, for some of the models, it does not discover the correct pressure-sensitive modeling features, leading to discrepancies of the yield surfaces in the $\pi _3$-direction. These discrepancies could be explained by a lack of data in the tensile or compressive regime, which may be alleviated by more complex experimental designs.

**Table 2:** Parameters of true and discovered *pressure-insensitive* material models for different noise levels $\sigma$ (in mm)

Benchmarks	$\theta _0$	$\theta _1$	$\theta _2$	$\theta _3$	$\theta _4$	$\eta$	$\overline{\eta }$	*R*	$p_a$
VM									
Truth	0.2400	0	0	0	0	0	0	NaN	$-\infty$
$\sigma = 0$	0.2400	0	0	0	0	0	0	NaN	$-\infty$
$\sigma = 10^{-4}$	0.2344	−0.0109	−0.0035	0	0	0	0	5.4531	−1.2426
$\sigma = 3 \cdot 10^{-4}$	0.1908	−0.0040	0	0	0.0012	1.1285	0.0162	NaN	$-\infty$
TR*									
Truth	0.2181	0	0.0095	0	0.0013	0	0	NaN	$-\infty$
$\sigma = 0$	0.2141	−0.0051	0.0072	0	0.0011	0	0	19.9210	−0.8855
$\sigma = 10^{-4}$	0.1932	−0.0188	0.0011	0	0	0.1422	0.0027	199.7617	−0.4641
$\sigma = 3 \cdot 10^{-4}$	0.1968	−0.0333	0.0055	−0.0010	0	0.1680	0.0257	2.1800	−1.7288
IV*									
Truth	0.1618	0.0300	0.0066	0.0021	0	0	0	NaN	$-\infty$
$\sigma = 0$	0.1612	0.0280	0.0053	0.0013	0	0.1268	0	NaN	$-\infty$
$\sigma = 10^{-4}$	0.1433	0.0249	0.0047	0.0011	0	0.9260	0.0245	NaN	$-\infty$
$\sigma = 3 \cdot 10^{-4}$	0.1416	0.0253	0.0045	0.0010	0	1.1749	0.0244	NaN	$-\infty$
MA*									
Truth	0.1954	−0.0332	0.0073	−0.0028	0.0012	0	0	NaN	$-\infty$
$\sigma = 0$	0.1948	−0.0322	0.0067	−0.0017	0	0.0109	0.0109	15.4624	−0.8655
$\sigma = 10^{-4}$	0.1688	−0.0280	0.0057	−0.0015	0	0.7270	0.0347	NaN	$-\infty$
$\sigma = 3 \cdot 10^{-4}$	0.1578	−0.0266	0.0052	−0.0015	0	1.8699	0.1215	1.0779	−1.8030

$\varvec{\theta }$ are in $\text {kN/}\text {mm}^2$. $p_a$ is in Pa

The value of $\eta$ determines the slope of the shear failure surface, and the value of $\bar{\eta }$ dictates the amount of dilatancy in the plastic deformations. For pressure-insensitive models, the slope of the surface in the $\pi _1$-$\pi _3$-plane should be $\infty$ ($\eta = 0$), and there is no dilatancy ($\bar{\eta } = 0$); for pressure-sensitive models, the value of $\bar{\eta }$ is usually much smaller than $\eta$ to avoid excessive dilatancy.^1^ From Table 1 we notice that the values of $\eta$ and $\bar{\eta }$ of DP* for $\sigma = 0$ and of DP*C for $\sigma = 0$ and $\sigma = 10^{-4}$ are exact. In most cases, for higher noise levels, smaller values of $\eta$ are obtained. Instead, the values of $\bar{\eta }$ are less influenced by the change in noise level. Besides, the $\bar{\eta }$ values of the IV*PC model are much smaller than the true ones, which may be caused by the absence of compression caps in the discovered solutions. Figure 7 shows that in the cases without noise ($\sigma = 0$) or with small noise ($\sigma = 10^{-4}$), the learned shapes of the shear failure surfaces are still very close to the true ones; when the noise level becomes larger ($\sigma = 3 \cdot 10^{-4}$), the discrepancy between the two surfaces is visible in the 3D plot. The pressure-sensitive terms vanish in the VM model for $\sigma = 0$ and $\sigma = 10^{-4}$, and in the TR* model for $\sigma = 0$. For higher noise, and even for the noiseless cases of the IV* and MA* models, the discovered values of $\eta$ and $\bar{\eta }$ are not zero, which means that with our dataset the penalization on $\eta$ in the cost function Eq. (14) is insufficient to completely eliminate the pressure-sensitive terms. However, these non-zero values are much smaller than those obtained for the pressure-sensitive material models.

The shape of the compression cap is defined by the two material parameters *R* and $p_a$. Although $\varvec{\theta }$ also affects the shape of the compression cap in the $\pi _1$–$\pi _2$-plane, here we focus on its shape in the $\pi _1$–$\pi _3$ and $\pi _2$–$\pi _3$-planes. For material models with a compression cap, the position of the learned compression cap should be as close as possible to that of the true model; for material models without a compression cap, the value of $p_a$ should approach $-\infty$ (i.e., the position of the compression cap should be as far as possible from the origin), and the value of *R* should go to 1 (the enforced lower bound for *R*, see “Material model library” section) because of the penalization in (Eq. (14)), since it has no influence on the cost value in this case. The results show that for models DP*C, IV*PC, and MA*PC we can always discover the compression cap at an accurate location. With increasing noise, the error between true and discovered values increases. The effect of noise on the solution is larger for *R* than for $p_a$; however, the influence of *R* on the shape of the yield surface is usually small as shown in Figs. 7c and 8b. Since the FE-generated dataset for the IV*PC model does not include a compression cap, the discovered model correctly shows no compression cap. In general, for pressure-sensitive models without compression cap, the discovered models correctly display no compression cap. However, for pressure-insensitive models, the compression caps disappear only for certain models and certain noise levels. On the other hand, since pressure-insensitive models never have a compression cap, it may be sufficient to detect that a model is pressure-insensitive (which can be done e.g. through the small value of $\eta$) and consequently ignore the results for *R* and $p_a$ as not relevant.

**Fig. 10:**
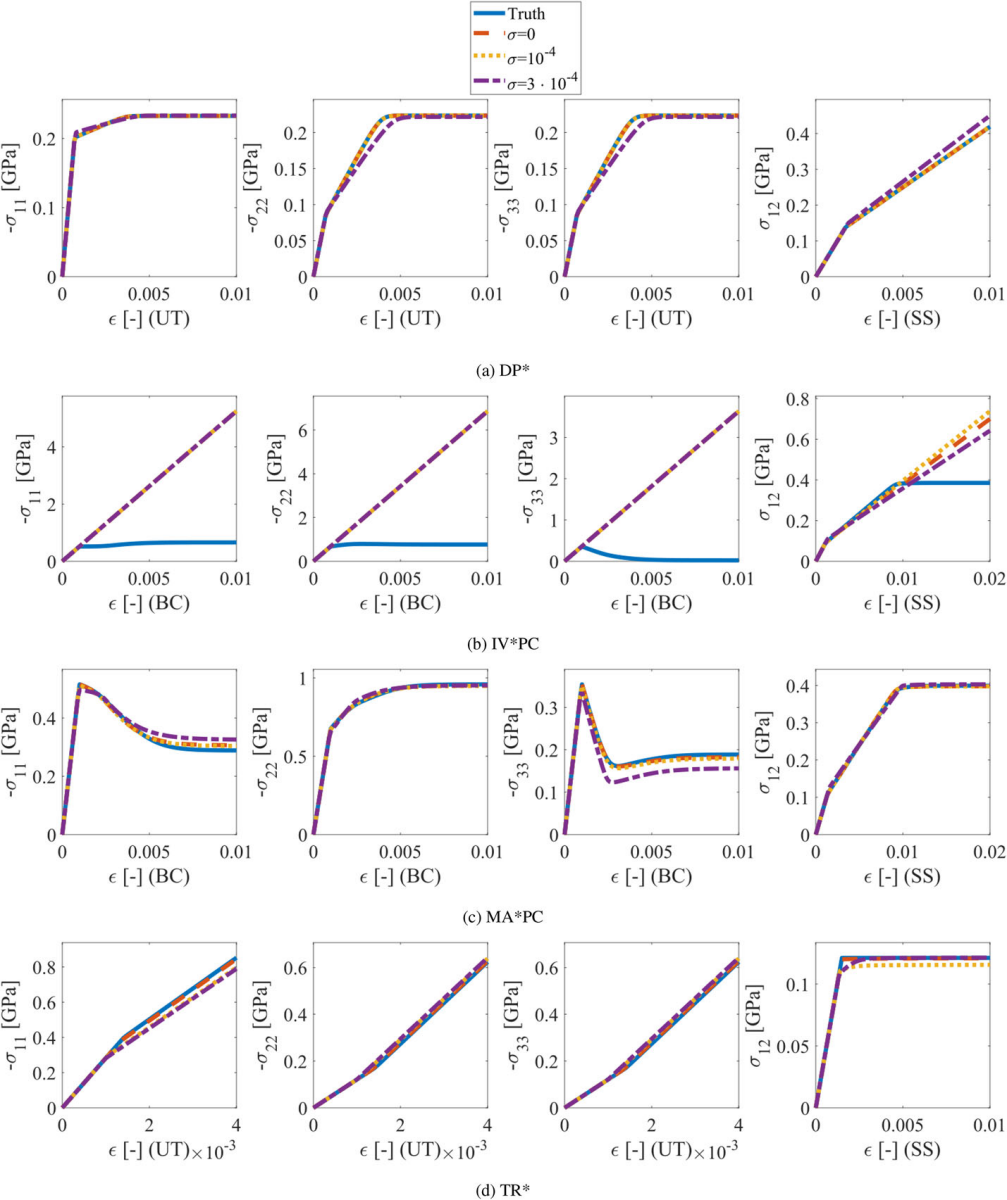
True and discovered material responses under uniaxial tension (UT), biaxial compression (BC) and simple shear (SS) with different noise levels $\sigma$ (in mm)

Finally, to evaluate whether the discovered material models accurately represent the material behavior of the ground truth models under loading conditions different from the ones used to obtain the data, we calculate the stress response of the true and discovered models for two distinct strain paths. For pressure-insensitive models and for pressure-sensitive models without compression cap, we select these strain paths as uniaxial tension (UT) and simple shear (SS), in order to test the stress behavior on the shear failure surfaces; for pressure-sensitive models with compression cap, we adopt biaxial compression (BC) and SS to show the response on both the shear failure and compression cap surfaces. These strain paths are given by

15 $$\begin{aligned} \begin{gathered} {\varvec{\varepsilon }^{\text {UT}} = \begin{bmatrix} \epsilon &  0 &  0\\ 0 &  0 &  0\\ 0 &  0 &  0\\ \end{bmatrix}, \quad \varvec{\varepsilon }^{\text {SS}} = \begin{bmatrix} 0 &  \epsilon &  0\\ \epsilon &  0 &  0\\ 0 &  0 &  0\\ \end{bmatrix}, \quad \varvec{\varepsilon }^{\text {BC}} = \begin{bmatrix} -\epsilon &  0 &  0\\ 0 &  -2 \epsilon &  0\\ 0 &  0 &  0\\ \end{bmatrix},} \end{gathered} \end{aligned}$$

where $\epsilon >0$ is a scalar deformation parameter. Figure 10 shows for the true and discovered models the stress components $\sigma _{11}$, $\sigma _{22}$, and $\sigma _{33}$ (for UT and BC) and $\sigma _{12}$ (for SS) as functions of $\epsilon$. Only the stress response for selected models is shown here, whereas the remaining plots are provided in Appendix G. The range of $\epsilon$ is selected such that the material state reaches the plastic regime for each material model, and so as to exceed the maximum deformation present in the training data. In general, Fig. 10 shows a good agreement between the true and discovered material responses, with discrepancies that, as expected, increase with the noise level. Larger discrepancies are observed for the model IV*PC under biaxial compression (as shown in Fig. 10b). For this model, EUCLID made a false-negative feature prediction for the compression cap. The absence of the compression cap explains why the resulting stress response is similar to that of pressure-sensitive models without the compression cap, i.e., the shear stress response of the DP* model shown in Fig. 10a.

## Conclusions

We have presented an extension of EUCLID to pressure-sensitive, non-associated plasticity. To this end, we introduce a model library by choosing an expressive parameterization of the yield function, comprising an arbitrarily shaped yield surface in the deviatoric plane, two pressure-sensitive terms for modeling the shear failure surface and the compression cap, and a similar yet different parameterization of the flow potential. The model library is chosen such that the yield function and the flow potential are smooth, in order to ease the usage of an elastic predictor-plastic corrector return mapping algorithm, and the material parameters are constrained such that the yield function and the flow potential are convex. We have shown that EUCLID is able to automatically discover interpretable pressure-sensitive and non-associated plasticity models from displacement and net reaction force data only, without using any stress data.

Some important issues remain open and should be addressed in future work. One point concerns the adequate coverage of the modeling space by the data, which is crucial for a successful discovery. To improve on this aspect, one option is to use results from more than one test. The other option is to optimize the specimen shape and topology to maximize the data richness. In both cases, quantitatively reliable indicators are needed to assess the coverage during the discovery procedure. A second important need, connected to the previous point, is to develop sound ways of assessing the quality of the discovered model, including quantification of its uncertainty, for real situations in which no ground truth is available. These and other aspects will be the subject of our forthcoming research.

## Figures and Tables

**Fig. 11:**
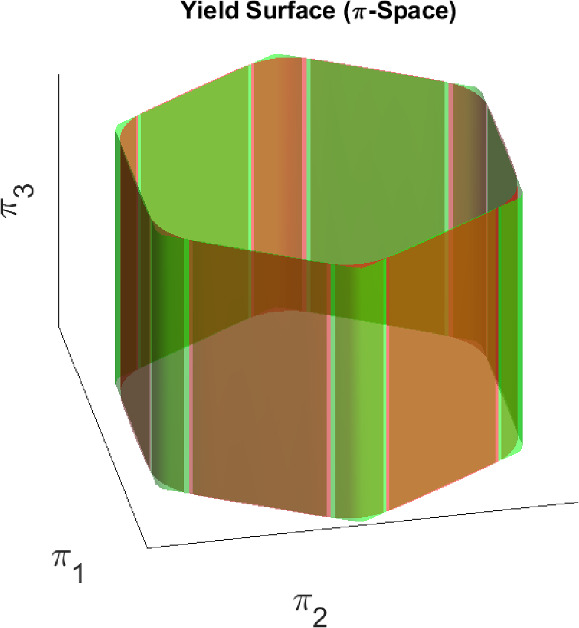
Smooth approximation (green) and convex smooth approximation (red) of the Tresca yield surface

**Fig. 12:**
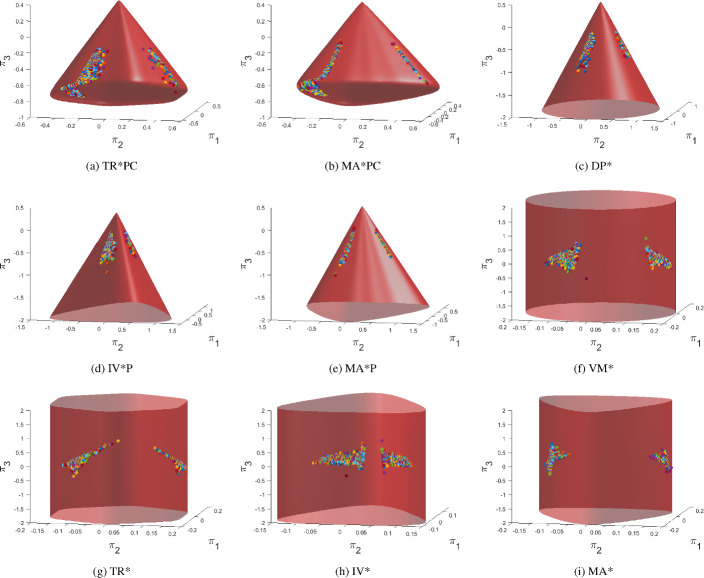
Remaining visualizations on the yield surface of the data from the biaxial compression test in Fig. 5

**Fig. 13:**
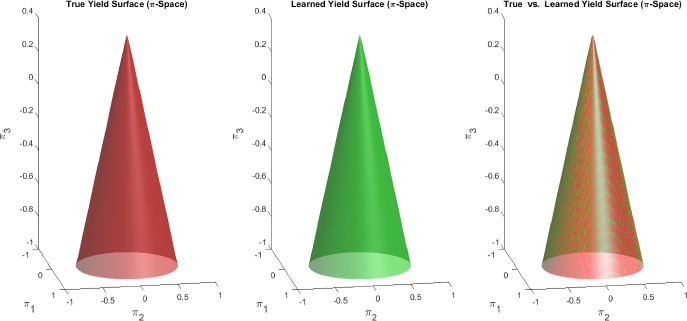
3D yield surface plots of the true and discovered DP* model for $\sigma = 0$

**Fig. 14:**
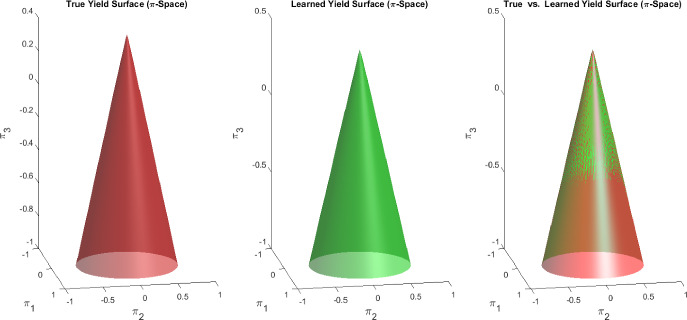
3D yield surface plots of the true and discovered DP* model for $\sigma = 10^{-4}$

**Fig. 15:**
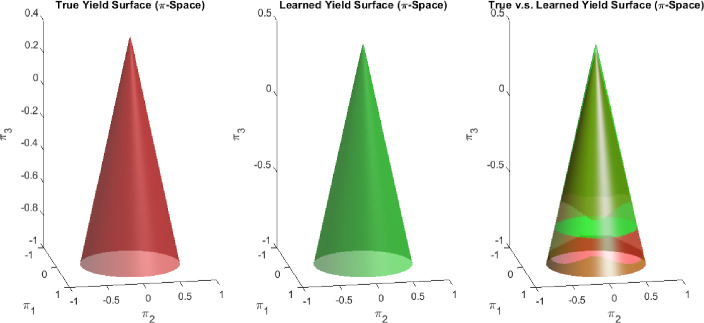
3D yield surface plots of the true and discovered DP* model for $\sigma = 3 \cdot 10^{-4}$

**Fig. 16:**
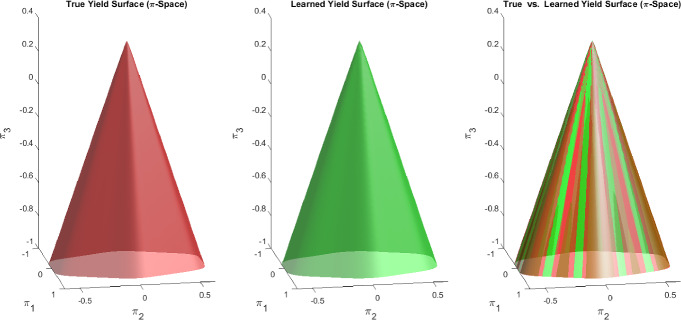
3D yield surface plots of the true and discovered TR*P model for $\sigma = 0$

**Fig. 17:**
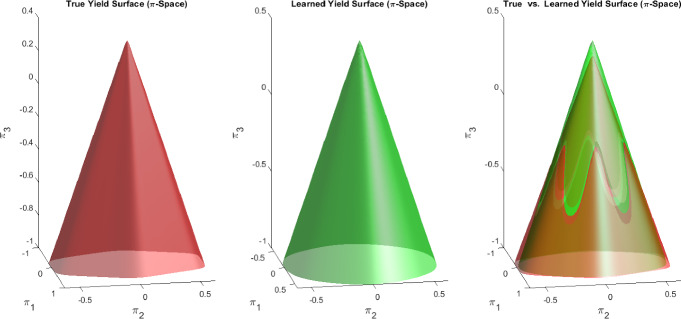
3D yield surface plots of the true and discovered TR*P model for $\sigma = 3 \cdot 10^{-4}$

**Fig. 18:**
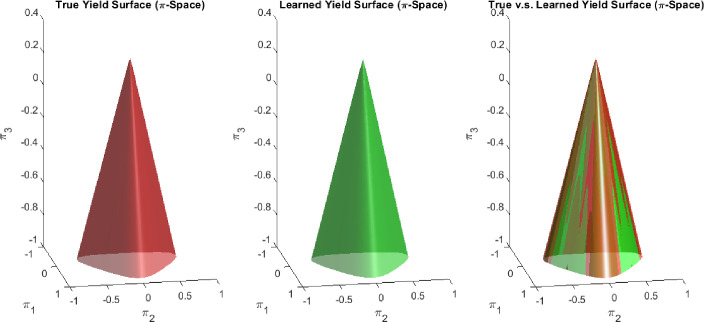
3D yield surface plots of the true and discovered IV*P model for $\sigma = 0$

**Fig. 19:**
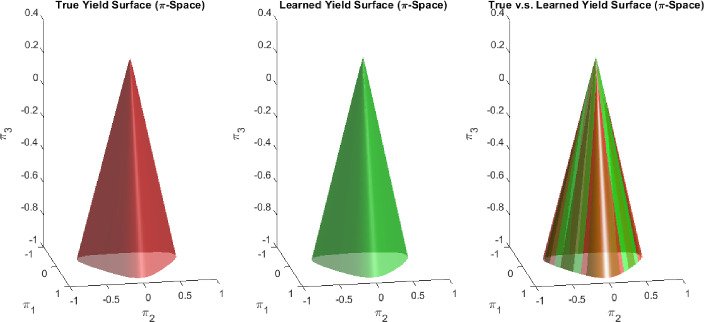
3D yield surface plots of the true and discovered IV*P model for $\sigma = 10^{-4}$

**Fig. 20:**
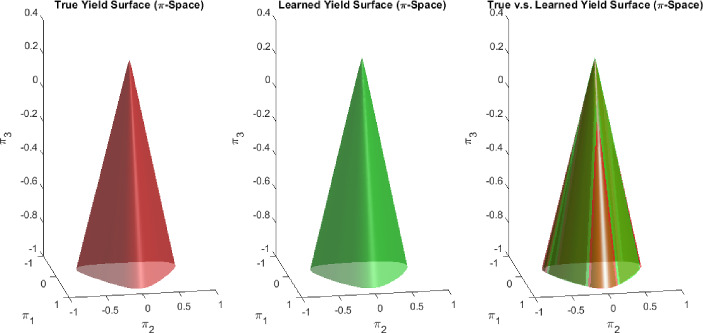
3D yield surface plots of the true and discovered IV*P model for $\sigma = 3 \cdot 10^{-4}$

**Fig. 21:**
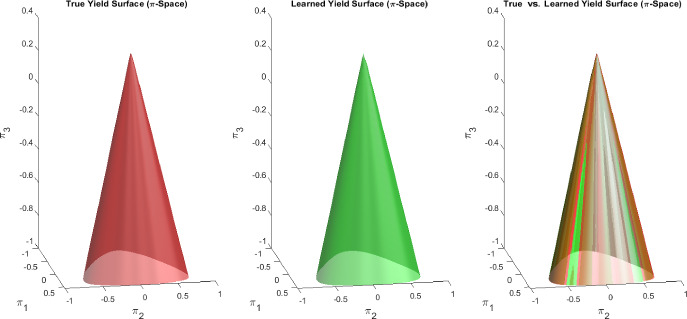
3D yield surface plots of the true and discovered MA*P model for $\sigma = 0$

**Fig. 22:**
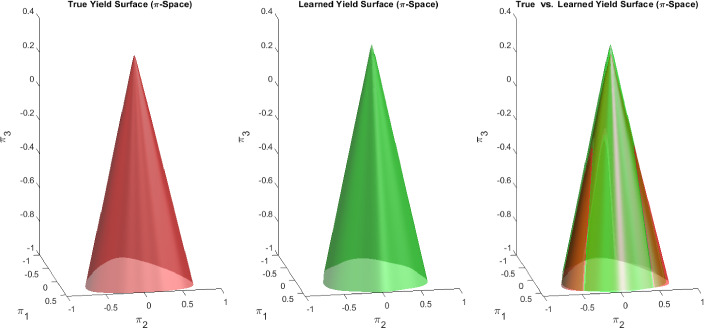
3D yield surface plots of the true and discovered MA*P model for $\sigma = 3 \cdot 10^{-4}$

**Fig. 23:**
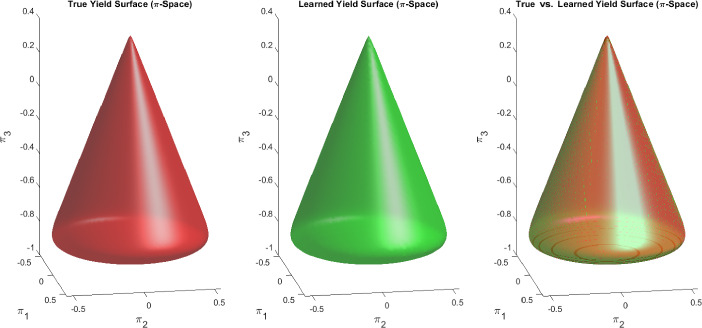
3D yield surface plots of the true and discovered DP*C model for $\sigma = 0$

**Fig. 24:**
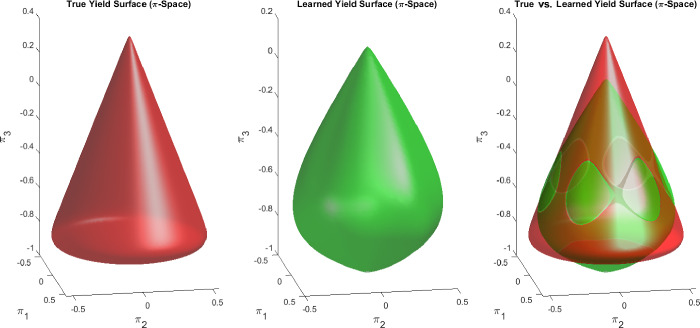
3D yield surface plots of the true and discovered DP*C model for $\sigma = 3 \cdot 10^{-4}$

**Fig. 25:**
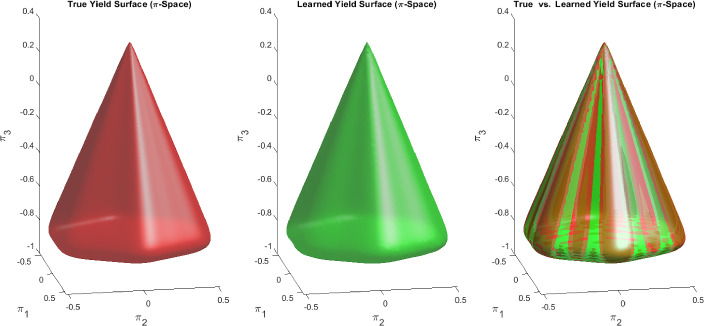
3D yield surface plots of the true and discovered TR*PC model for $\sigma = 0$

**Fig. 26:**
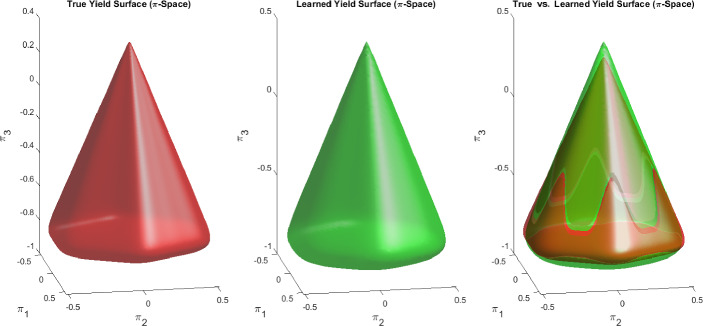
3D yield surface plots of the true and discovered TR*PC model for $\sigma = 3 \cdot 10^{-4}$

**Fig. 27:**
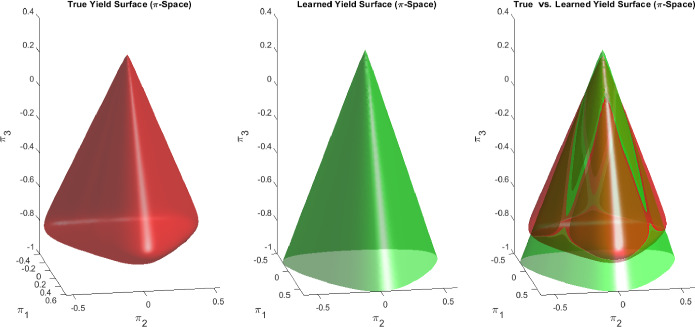
3D yield surface plots of the true and discovered IV*PC model for $\sigma = 0$

**Fig. 28:**
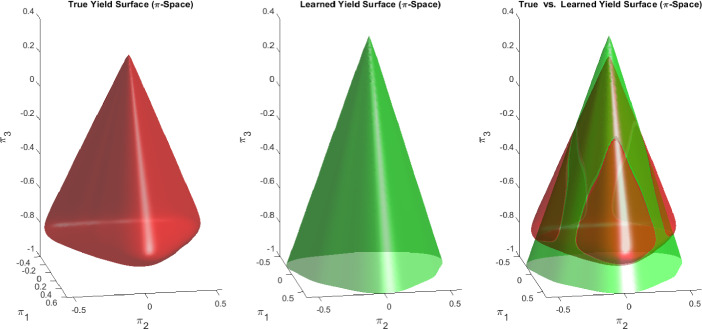
3D yield surface plots of the true and discovered IV*PC model for $\sigma = 3 \cdot 10^{-4}$

**Fig. 29:**
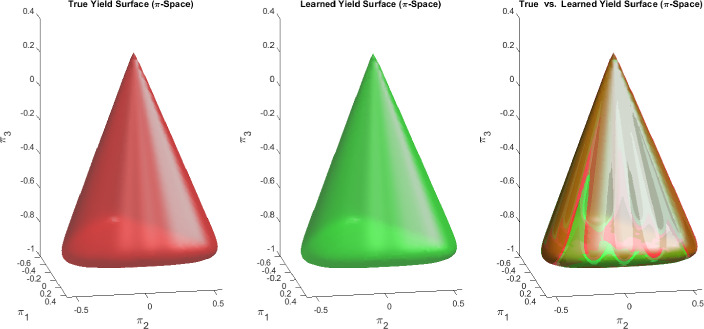
3D yield surface plots of the true and discovered MA*PC model for $\sigma = 0$

**Fig. 30:**
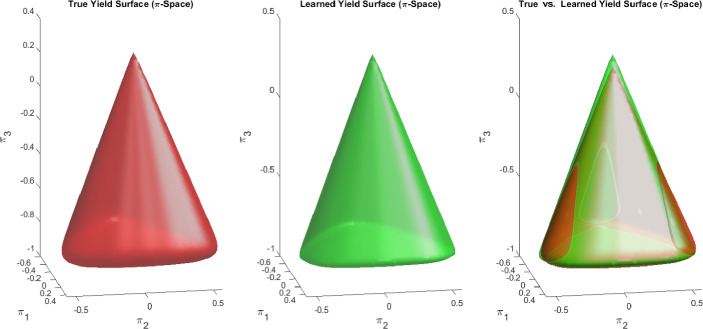
3D yield surface plots of the true and discovered MA*PC model for $\sigma = 3 \cdot 10^{-4}$

**Fig. 31:**
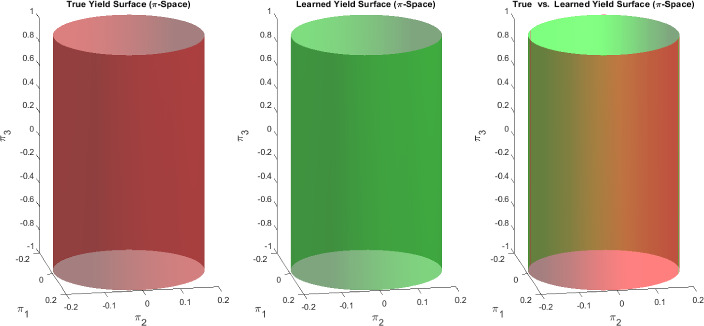
3D yield surface plots of the true and discovered VM model for $\sigma = 0$

**Fig. 32:**
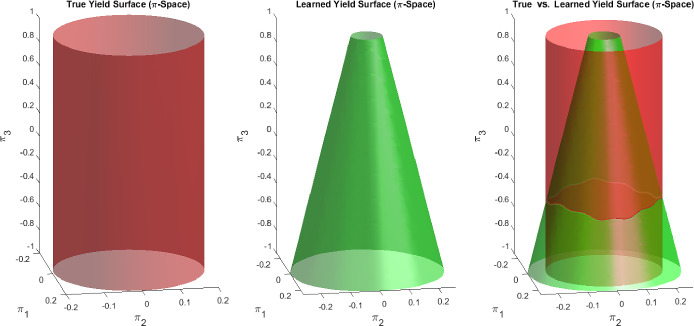
3D yield surface plots of the true and discovered VM model for $\sigma = 3 \cdot 10^{-4}$

**Fig. 33:**
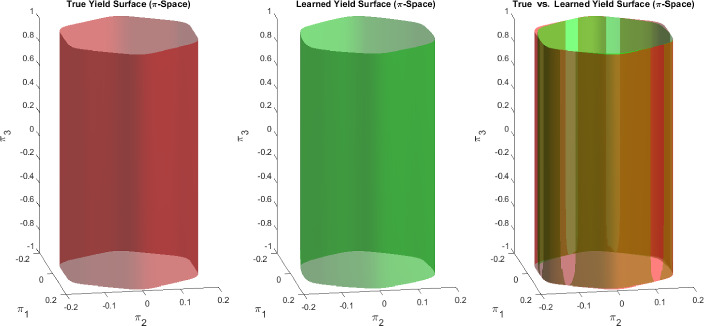
3D yield surface plots of the true and discovered TR* model for $\sigma = 0$

**Fig. 34:**
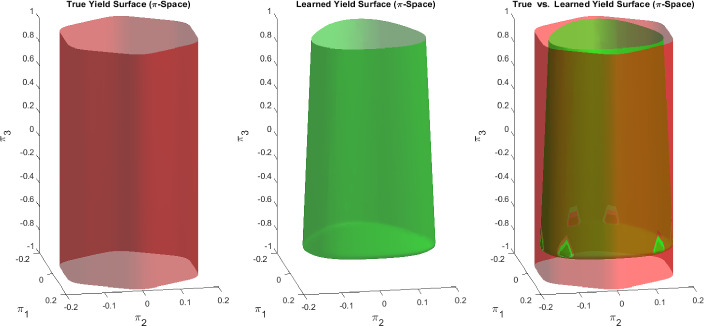
3D yield surface plots of the true and discovered TR* model for $\sigma = 10^{-4}$

**Fig. 35:**
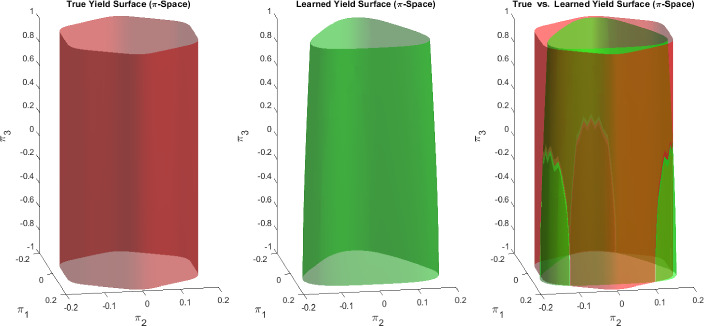
3D yield surface plots of the true and discovered TR* model for $\sigma = 3 \cdot 10^{-4}$

**Fig. 36:**
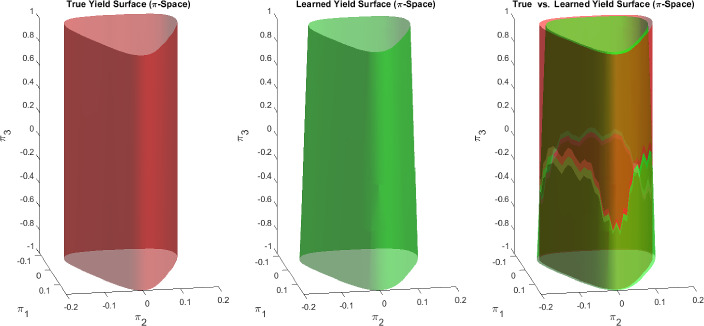
3D yield surface plots of the true and discovered IV* model for $\sigma = 0$

**Fig. 37:**
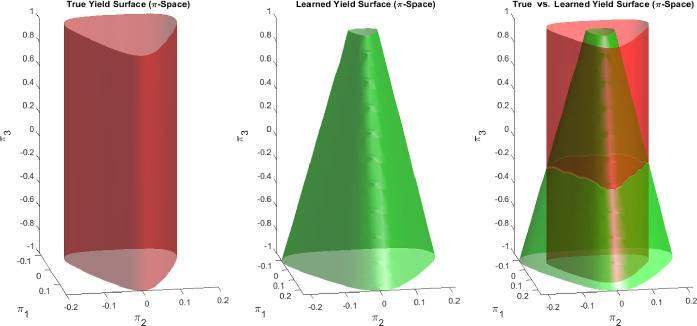
3D yield surface plots of the true and discovered IV* model for $\sigma = 3 \cdot 10^{-4}$

**Fig. 38:**
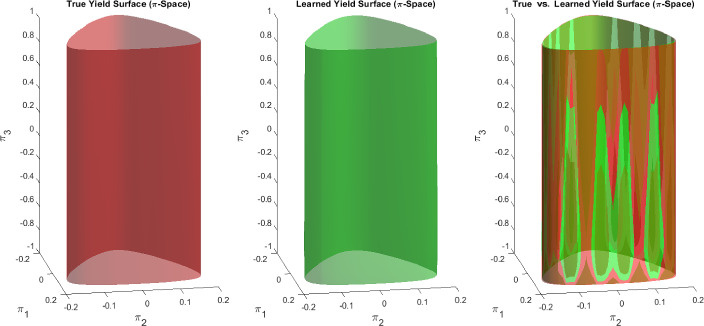
3D yield surface plots of the true and discovered MA* model for $\sigma = 0$

**Fig. 39:**
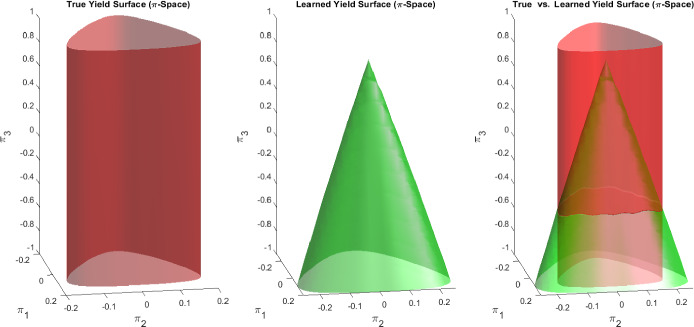
3D yield surface plots of the true and discovered MA* model for $\sigma = 3 \cdot 10^{-4}$

**Fig. 40:**
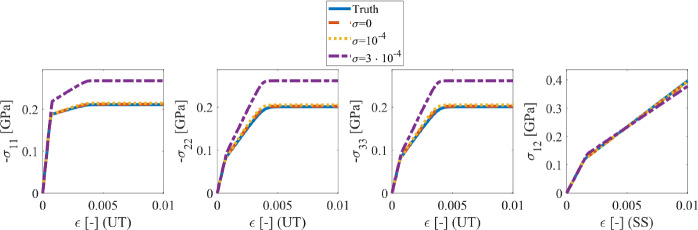
Stress response of TR*P model under uniaxial tension (UT) and simple shear (SS) with different noise levels $\sigma$ (in mm)

**Fig. 41:**
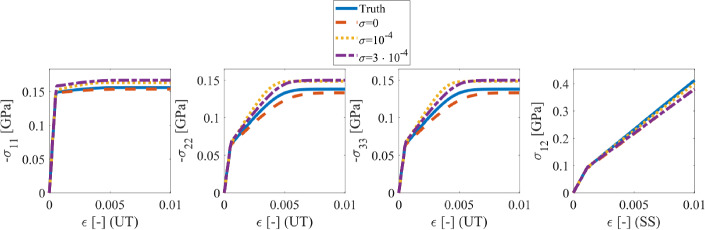
Stress response of IV*P model under uniaxial tension (UT) and simple shear (SS) with different noise levels $\sigma$ (in mm)

**Fig. 42:**
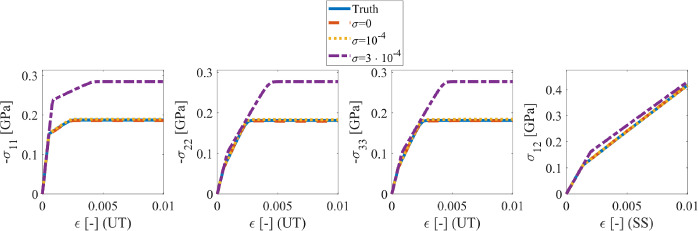
Stress response of MA*P model under uniaxial tension (UT) and simple shear (SS) with different noise levels $\sigma$ (in mm)

**Fig. 43:**
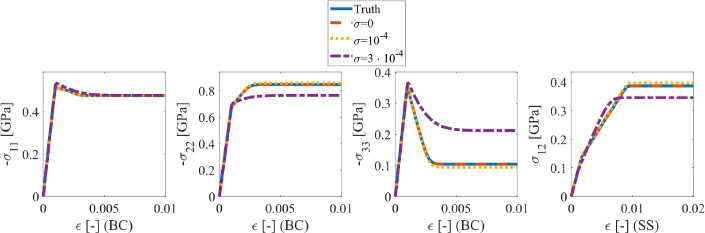
Stress response of DP*C model under biaxial compression (BC) and simple shear (SS) with different noise levels $\sigma$ (in mm)

**Fig. 44:**
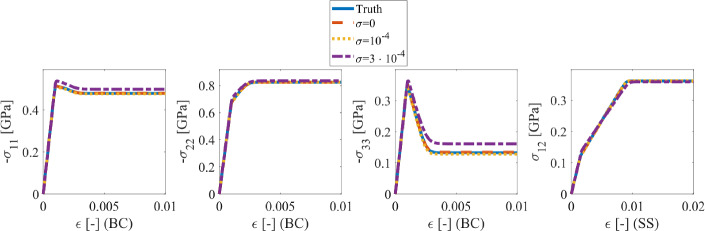
Stress response of TR*PC model under biaxial compression (BC) and simple shear (SS) with different noise levels $\sigma$ (in mm)

**Fig. 45:**
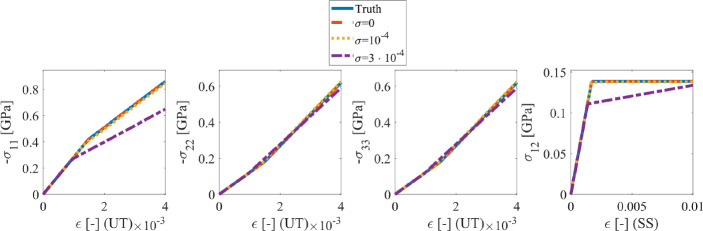
Stress response of VM* model under uniaxial tension (UT) and simple shear (SS) with different noise levels $\sigma$ (in mm)

**Fig. 46:**
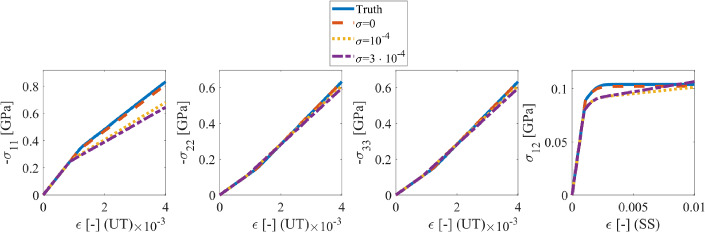
Stress response of IV* model under uniaxial tension (UT) and simple shear (SS) with different noise levels $\sigma$ (in mm)

**Fig. 47:**
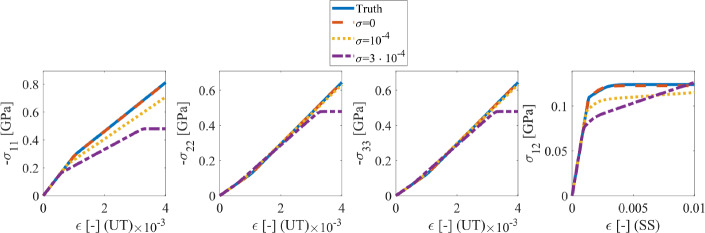
Stress response of MA* model under uniaxial tension (UT) and simple shear (SS) with different noise levels $\sigma$ (in mm)

## Data Availability

The codes and data generated during the current study will be uploaded to https://euclid-code.github.io/ upon publication.

## Appendix A: Elastic predictor-plastic corrector return mapping algorithm

The local problem is solved with the return mapping algorithm for computing the stress and updating the history variables. Assuming perfect plasticity, the only history variable that needs to be updated is the plastic strain. In the plane-strain problem, the plastic strain tensor contains four components

A.1 $$\begin{aligned} \begin{gathered} \varvec{\varepsilon }_p = \begin{bmatrix} (\varepsilon _p)_{11} &  (\varepsilon _p)_{12} &  0\\ (\varepsilon _p)_{12} &  (\varepsilon _p)_{22} &  0\\ 0 &  0 &  (\varepsilon _p)_{33}\\ \end{bmatrix}. \end{gathered} \end{aligned}$$

Besides, the elastic strain component $(\varvec{\varepsilon }_e)_{33}$ is no longer zero as in linear elastic problems, since

A.2 $$\begin{aligned} \begin{gathered} \varepsilon _{33} = (\varepsilon _e)_{33} + (\varepsilon _p)_{33} = 0. \end{gathered} \end{aligned}$$

The update of stress states and history variables is governed by the Karush-Kuhn-Tucker loading/unloading conditions (Eq. (2)) and the plastic evolution law (Eq. (1)). As a result, the return mapping algorithm consists in solving a system of nonlinear equations at each time step *t*, and with an implicit Euler time discretization we obtain

A.3 $$\begin{aligned} \begin{aligned} f^t&= 0,\\ \varvec{\varepsilon }^t_p&= \varvec{\varepsilon }^{t-1}_p + \Delta \gamma ^t \frac{\partial \Psi ^t(\varvec{\sigma })}{\partial \varvec{\sigma }^t}, \end{aligned} \end{aligned}$$

where $f^t$ and $\varvec{\varepsilon ^t_p}$ are the yield function and the plastic strain at time step t, $\Delta \gamma$ is the increment of the internal variable $\gamma$, and $\varvec{\varepsilon }^{t-1}_p$ is the plastic strain at the previous time step.

We choose the Newton–Raphson method to solve Eq. (A.3). Hence, we need to calculate the first derivative of the yield function *f* and the first and second derivative of the plastic potential $\Psi$ with respect to the stress $\varvec{\sigma }$. In [18], the first and second derivatives of $f_r=\sqrt{\frac{3}{2}}r$ and $f_\alpha = \sum _{i=0}^{n_f}{\theta _i \cos (3i\alpha )}$ with respect to $\varvec{\sigma }$ are provided, and thus they are not derived here.

To make the expressions shorter, we set

A.4 $$\begin{aligned} A = \eta ^2 f_\alpha ^2 a^2 + f_r^2, \end{aligned}$$

which is the term under the square root in Eq. (3). Then, the first and second derivatives of A can be written as

A.5 $$\begin{aligned} \begin{gathered} \frac{\partial A}{\partial \varvec{\sigma }} = 2 \eta ^2 a^2 f_\alpha \frac{\partial f_\alpha }{\partial \varvec{\sigma }} + 2 f_r \frac{\partial f_r}{\partial \varvec{\sigma }}, \\ \frac{\partial ^2 A}{\partial \varvec{\sigma }^2} = 2 \eta ^2 a^2 \left( \frac{\partial f_\alpha }{\partial \varvec{\sigma }} \otimes \frac{\partial f_\alpha }{\partial \varvec{\sigma }} + f_\alpha \frac{\partial ^2 f_\alpha }{\partial \varvec{\sigma }^2}\right) + 2\left( \frac{\partial f_r}{\partial \varvec{\sigma }} \otimes \frac{\partial f_r}{\partial \varvec{\sigma }} + f_r \frac{\partial ^2 f_r}{\partial \varvec{\sigma }^2}\right) .\\ \end{gathered} \end{aligned}$$

Compared to the yield function in [18], our yield function displays another term that is related to $\varvec{\sigma }$, namely, the hydrostatic pressure term $\left( \frac{1}{\sqrt{3}}\pi _3\right)$. The derivatives of $\left( \frac{1}{\sqrt{3}}\pi _3\right)$ with respect to $\varvec{\sigma }$ are written as

A.6 $$\begin{aligned} \frac{\partial \left( \frac{1}{\sqrt{3}}\pi _3\right) }{\partial \varvec{\sigma }} = \frac{1}{3}\varvec{I}, \quad \frac{\partial ^2 \left( \frac{1}{\sqrt{3}}\pi _3\right) }{\partial \varvec{\sigma }^2} = 0, \end{aligned}$$

where $\varvec{I}$ is the second-order unit tensor.

As a result, the derivatives of the yield function can be written as

A.7 $$\begin{aligned} \begin{aligned} \frac{\partial f}{\partial \varvec{\sigma }}&= \frac{1}{2} A^{-\frac{1}{2}} \frac{\partial A}{\partial \varvec{\sigma }} - \left( 1 - \frac{1}{\sqrt{3}}\eta \pi _3 - e^{- R (\frac{1}{\sqrt{3}}\pi _3 - p_a)}\right) \frac{\partial f_\alpha }{\partial \varvec{\sigma }} \\&\quad + \left( \eta - R e^{- R (\frac{1}{\sqrt{3}}\pi _3 - p_a)}\right) f_\alpha \frac{\partial \left( \frac{1}{\sqrt{3}}\pi _3\right) }{\partial \varvec{\sigma }},\\ \frac{\partial ^2 f}{\partial \varvec{\sigma }^2}&= -\frac{1}{4} A^{-\frac{3}{2}} \frac{\partial A}{\partial \varvec{\sigma }} \otimes \frac{\partial A}{\partial \varvec{\sigma }} + \frac{1}{2} A^{-\frac{1}{2}} \frac{\partial ^2 A}{\partial \varvec{\sigma }^2} - \left( 1 - \frac{1}{\sqrt{3}}\eta \pi _3 - e^{- R (\frac{1}{\sqrt{3}}\pi _3 - p_a)}\right) \frac{\partial ^2 f_\alpha }{\partial \varvec{\sigma }^2}\\&\quad + 2\left( \eta - R e^{- R (\frac{1}{\sqrt{3}}\pi _3 - p_a)}\right) \frac{\partial f_\alpha }{\partial \varvec{\sigma }} \otimes \frac{\partial \left( \frac{1}{\sqrt{3}}\pi _3\right) }{\partial \varvec{\sigma }} \\&\quad + R^2 e^{- R (\frac{1}{\sqrt{3}}\pi _3 - p_a)}f_\alpha \frac{\partial \left( \frac{1}{\sqrt{3}}\pi _3\right) }{\partial \varvec{\sigma }} \otimes \frac{\partial \left( \frac{1}{\sqrt{3}}\pi _3\right) }{\partial \varvec{\sigma }}, \end{aligned} \nonumber \\ \end{aligned}$$

and the derivatives of the plastic potential $\Psi$ can be straightforwardly obtained by exchanging $\eta$ with $\overline{\eta }$ in Eq. (A.7).

In addition to updating the plastic strain and stress states, as part of the local problem we also need to compute the elasto-plastic consistent tangent modulus, which is needed for the forward FE problem. Here, we apply the central finite difference method. In future works, one may also leverage automatic differentiation which is commonly used for derivative calculations in the field of machine learning.

## Appendix B: Convexity constraints

In the following, we derive the convexity constraint for the yield surface and comment on the numerical treatment of the constraint. If only $\theta _0 \ne 0$ in $\varvec{\theta }$, the yield surface can be imagined as a 3D surface generated by rotating a triangle-like 2D curve in the $\pi _1$–$\pi _3$-plane along the $\pi _3$-axis. When $\theta _i \ne 0$ $(i=1,2,...)$, while rotating, the 2D curve is also stretched or compressed along the direction of the Lode radius *r* depending on $\varvec{\theta }$. For example, as qualitatively shown in the second figure of the first row of Fig. 4, while rotating, the 2D curve needs to be stretched when it reaches the ridge of the surface; and it needs to be compressed when it is between the two ridges. It is obvious that the triangle-like 2D figure in the $\pi _1$–$\pi _3$-plane is always convex. Therefore, we only need to force the rotation path in the $\pi _1$–$\pi _2$-plane to be convex, i.e., it suffices to consider convexity of the yield surface for $\pi _3=0$. Further, without loss of generality, it suffices to consider convexity of the yield surface for $a=0$, $p_a=-\infty$.

The yield function is then given by (see Eq. (3))

B.1 $$\begin{aligned} f(r,\alpha )=\sqrt{\frac{3}{2}}r-h(\alpha ), \quad \text {with} \quad h(\alpha ) = \sum _{i=0}^{n_f}{\theta _i \cos (3i\alpha )}. \end{aligned}$$

The convexity requirement is fulfilled if and only if the hessian matrix $H_{ij} = \frac{\partial ^2 f}{\partial \sigma _i \partial \sigma _j}$ of the yield function is positive semi-definite. In the following, we derive from this requirement the constraint on the material parameters $\varvec{\theta }$ (see Eq. (7)). For similar derivations, we refer to [49].

The Hessian matrix can be written as

B.2 $$\begin{aligned} \begin{aligned} \varvec{H}&= \frac{\partial ^2 f}{\partial \varvec{\sigma }^2}\\&=\sqrt{\frac{3}{2}} \frac{\partial ^2 r}{\partial \varvec{\sigma }^2} - \frac{\partial ^2 h}{\partial \alpha ^2} \frac{\partial ^2 \alpha }{\partial \varvec{\sigma }^2}, \end{aligned} \end{aligned}$$

where the first term $\frac{\partial ^2 r}{\partial \varvec{\sigma }^2}$ is positive semi-definite [49]. Since $r>0$, we can multiply by *r* both sides of the equation

B.3 $$\begin{aligned} \begin{gathered} r \varvec{H}=\sqrt{\frac{3}{2}} r \frac{\partial ^2 r}{\partial \varvec{\sigma }^2} - \frac{\partial ^2 h}{\partial \alpha ^2} r \frac{\partial ^2 \alpha }{\partial \varvec{\sigma }^2}, \end{gathered} \end{aligned}$$

without changing the positive semi-definiteness of $\varvec{H}$. With reference to [49], we obtain

B.4 $$\begin{aligned} \begin{gathered} r \frac{\partial ^2 \alpha }{\partial \varvec{\sigma }^2} = \frac{\partial ^2 r}{\partial \varvec{\sigma }^2}. \end{gathered} \end{aligned}$$

Then, we obtain

B.5 $$\begin{aligned} \begin{gathered} r \varvec{H}= \left( \sqrt{\frac{3}{2}} r - \frac{\partial ^2 h}{\partial \alpha ^2}\right) \frac{\partial ^2 r}{\partial \varvec{\sigma }^2}. \end{gathered} \end{aligned}$$

Since the yield surface is obtained by $f = \sqrt{\frac{3}{2}}r-h(\alpha ) = 0$, we have $\sqrt{\frac{3}{2}}r = h(\alpha )$. As a result, $\varvec{H}$ is positive semi-definite if and only if $h(\alpha ) -\frac{\partial ^2 h}{\partial \alpha ^2} \ge 0$. Finally, the convexity constraint can be written as

B.6 $$\begin{aligned} \ \begin{gathered} \text {f is convex} \Longleftrightarrow h(\alpha ) -\frac{\partial ^2 h}{\partial \alpha ^2} \ge 0, \quad \forall \alpha \in [0,\frac{\pi }{3}], \end{gathered} \end{aligned}$$

where the range of $\alpha$ for the constraint is determined by the symmetry properties of the yield function. Substituting *h* leads to

B.7 $$\begin{aligned} \begin{gathered} \text {f is convex} \Longleftrightarrow \theta _0 + \sum _{i=0}^{n_f}(1 + 9 i^2)\theta _i \cos (3 i \alpha ) \ge 0, \quad \forall \alpha \in [0,\frac{\pi }{3}]. \end{gathered} \end{aligned}$$

Since it is not feasable to check the convexity constraints at every single value of $\alpha \in [0,\frac{\pi }{3}]$, during the optimization process the original convexity constraint is relaxed to a system of inequalities as follows

B.8 $$\begin{aligned} \begin{gathered} \theta _0 + \sum _{i=0}^{n_f}(1 + 9 i^2)\theta _i \cos (3 i \alpha _j) \ge 0, \quad \alpha _j \in \left\{ \frac{\pi }{60},\frac{2\pi }{60},...,\frac{20\pi }{60} \right\} . \end{gathered} \end{aligned}$$

## Appendix C: Convex and smooth approximation of yield surfaces

As described in “Data generation” section, the original Tresca, Ivlev, and Mariotte models are non-smooth. The Fourier series (Eq. (3)) can be used to find smooth approximations of these models. However, due to the oscillatory nature of the features in the Fourier series, it is very likely that the resulting yield surface is non-convex in some regions, if the parameters in the Fourier series are not constrained. This effect is very small and barely visible when plotting the yield surface (see the green yield surface in Fig. 11), but it can lead to convergence issues of the return mapping algorithm. To avoid this, we here constrain the parameters in the Fourier series such that the resulting smooth approximation of the yield surface is convex. This slightly reduces the quality of the approximation (see the red yield surface in Fig. 11), but guarantees convexity and robustness of the return mapping algorithm.

## Appendix D: Default parameters and hyperparameters for FE data generation and EUCLID

Tables 3 and 4 provide lists of default parameters and hyperparameters for FE data generation and EUCLID, respectively.

**Table 3:** Default parameters and hyperparameters for FE data generation

FE data generation			
Parameter	Notation	Value	Unit
Number of nodes in mesh	$n_n$	2179	–
Number of reaction force constraints	$n_{\beta }$	2	–
Number of time steps	$n_t$	400	–
Loading parameter	$\delta$	0.1	mm
Young’s modulus	*E*	210	kN/mm$^2$
Poisson’s ratio	$\nu$	0.3	–
Displacement noise standard deviation	$\sigma$	$\{0, 10^{-4}, 3 \cdot 10^{-4}\}$	mm
Denoising moving-window length	–	20	–

**Table 4:** Default parameters and hyperparameters for EUCLID

EUCLID			
Parameter	Notation	Value	Unit
Number of Fourier features	$n_f + 1$	5	–
Coefficient for reaction force balance	$\lambda _{r}$	100	–
Coefficient for $\ell _1$ regularization	$\lambda _{p}$	$\{2^i:i=-9,...,20\}$	mm$^{2}$
Number of random initial guesses	$n_g$	100	–
Threshold for *C*	$C^{th}$	$1.0001 \cdot C^{min}$	*kN*
Threshold for $\varvec{\theta }$	$\theta ^{th}$	$0.005 \cdot \theta _0$	kN/mm$^2$
Threshold for $\eta$	$\eta ^{th}$	0.01	mm$^2$/kN
Threshold for $\bar{\eta }$	$\bar{\eta }^{th}$	0.01	mm$^2$/kN
Threshold for $$p_a$$	$$p_a^{th}$$	$$-20 $$	kN/mm$$^2$$

## Appendix E: Plots of the dataset on the yield surfaces

The plots of the dataset on the yield surfaces not shown in Fig. 6 are displayed in this section.

## Appendix F: Plots of discovered yield surfaces

The plots of the discovered and true yield surfaces not shown in “Results” section are displayed in this section (Figs.  12, 13, 14, 15, , , , , , , , , , , , , , , , 31, 32, 33, 34, 35, 36, 37, 38, 39).

## Appendix G: Plots of stress curves

The plots of the stress responses not shown in “Results” section are displayed in this section (Figs. 40, 41, 42, 43, 44, 45, 46, 47).

## Footnotes

1 Practically, the value of $\bar{\eta }$ cannot be too small to avoid problems with the convergence of the local problem. In the true models used for data generation in this work, we set $\bar{\eta } = \frac{1}{5}\eta$.
